# Radical cascades using enantioenriched 7-azabenzonorbornenes and their applications in synthesis

**DOI:** 10.3762/bjoc.4.38

**Published:** 2008-10-24

**Authors:** David M Hodgson, Leonard H Winning

**Affiliations:** 1 Department of Chemistry, Chemistry Research Laboratory, University of Oxford, Mansfield Road, Oxford, OX1 3TA, UK; Fax: +44(1865) 285002

**Keywords:** asymmetric synthesis, deoxygenation, radicals, rearrangements, tandem reactions

## Abstract

Tandem deoxygenation–neophyl-type radical rearrangement–electrophile trapping using xanthates from 7-azabenzonorbornadienes gives 3-*exo*-substituted 2-aza-5,6-benzonorbornenes, which in some cases undergo isomerisation to (aminomethyl)indenes. The starting xanthates are accessible in good yields and high enantiomeric ratios via asymmetric hydroboration of (aryne/pyrrole-derived) 7-azabenzonorbornadienes. Oxidation (using RuO_4_) and Birch reduction of the 2-aza-5,6-benzonorbornenes provide access to substituted pyrrolidines and tetrahydroindenes, respectively.

## Introduction

Carbon-centred radicals have been shown to be useful intermediates in organic chemistry with widespread applications in synthesis [1–6]. Significant advantages can be gained by the use of radical intermediates: for example, despite their highly reactive nature, radical intermediates can be generated under mild conditions without the need for strongly acidic or basic environments [7]. Furthermore, an understanding of radical chain reactions has enabled radical-based methods to be applied to the synthesis of complex targets [8–9], and routes using radical intermediates can readily be considered during retrosynthetic planning [10].

**Scheme 1:**
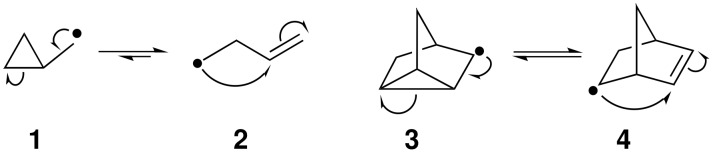
Cyclopropylmethyl–homoallyl and nortricyclyl–norbornenyl radical systems.

The cyclopropylmethyl radical (**1**) is known to ring-open to homoallyl radical (**2**) with a rate of 1.2 × 10^8^ s^−1^ at 37 °C (Scheme 1) [11].

Substituted cyclopropylmethyl–homoallylic radical systems are found embedded in a range of more complex substrates, such as those based upon the bicyclo[2.2.1]heptenyl (norbornenyl) framework. In these latter systems, nortricyclyl radical **3** can undergo reversible ring-opening to norbornenyl radical **4**; treatment of nortricyclyl or norbornenyl bromide with Bu_3_SnH and AIBN is known to lead to the same (approximately equal) mixture of nortricyclane and norbornene [12–13]. The final product distribution in these systems is found to be heavily dependent on the reactant concentrations as well as on the structure of the substrate. In synthetic applications it is therefore important to be able to bias the rearrangement in favour of the desired product, which is most conveniently achieved by the incorporation of one or more structural directing effect(s). Research in our laboratory has focussed on using the potential dative stabilising effect of an α-nitrogen in the product-producing radical to direct homoallylic radical rearrangements for use in azacycle synthesis [14].

The utility of nitrogen-directed radical rearrangements in the 7-azanorbornene system has been reported previously in relation to the synthesis of a variety of biologically-relevant targets, including epibatidine analogues [15–16], kainic acid [17–18] and ibogamine [19]. In these studies, the radical step has mainly been carried out in the presence of a relatively fast radical reductant (e.g. Bu_3_SnH). More recently, investigations have centred on the use of (TMS)_3_SiH as a slower hydrogen atom donor, with a view to affecting tandem radical generation–rearrangement–electrophile trapping for the rapid enhancement of molecular complexity. Furthermore, in the structurally similar system created by the formal fusion of the norbornenyl skeleton with an aromatic ring, the ‘nortricyclyl’ radical is delocalised in the originally aromatic π-system. Ring-opening of this ‘nortricyclyl’ radical species can lead to an overall 1,2-aryl (neophyl) migration [20]. We have previously communicated our initial findings in the application of tandem radical cascades toward the 2-aza-5,6-benzonorbornenyl system [21]. We now present our wider investigations of this system and its synthetic utility.

## Results and Discussion

**Scheme 2:**
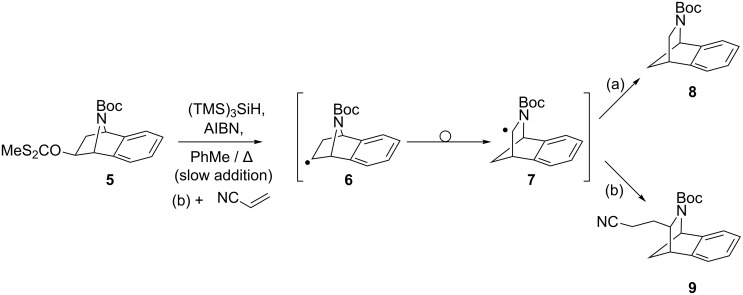
Deoxygenation-rearrangement-electrophile trapping.

Chatgilialoglu and co-workers have previously shown that (TMS)_3_SiH-mediated xanthate deoxygenations can be performed in tandem with electrophile trapping [22–23]. We were encouraged that the best yields of deoxygenated, trapped product relative to directly reduced product were achieved under conditions [refluxing toluene, thermal initiation by AIBN] similar to those we had previously reported for the tandem deoxygenation–rearrangement–reduction of 7-azabenzonorbornenyl xanthates (e.g. **5** to **8**, Scheme 2), although the work in our laboratory had employed a syringe pump in order to achieve a slow rate of addition of the radical initiator and reductant [21,24]. Pleasingly, combining these procedures, *i.e.* performing a slow addition of AIBN, (TMS)_3_SiH (1.5 equiv) and acrylonitrile (1.5 equiv) to a 0.03 M solution of xanthate **5** [24–25] in refluxing toluene, resulted in the formation of rearranged-trapped azacycle **9**, in 77% yield (Scheme 2), and exclusively as the *exo*-isomer (for details see Supporting Information File 1).

**Figure 1:**
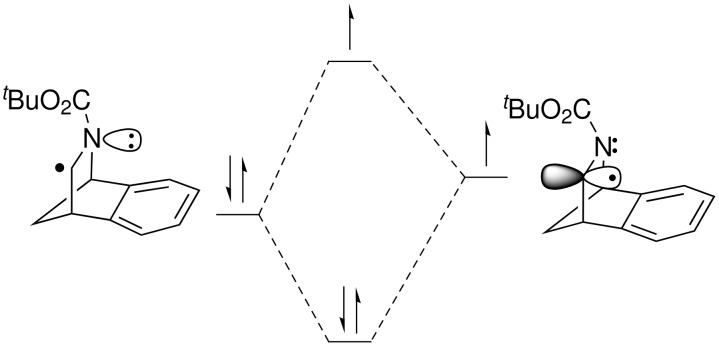
Radical SOMO/α-nitrogen lone-pair interaction in the rearranged radical **7**.

In contrast to Chatgilialoglu’s work, products from reduction of radicals **6** or **7** were not observed, nor was the product of electrophile trapping by the unrearranged radical **6** detected. By-products isolated from the reaction all showed large Me-Si signals in their ^1^H NMR spectra, suggesting that the (TMS)_3_Si radical had added to the system; however the structures of these compounds could not be determined. The selectivity that is observed for the product azacycle **9** can be rationalised in terms of the higher energy SOMO and therefore higher nucleophilicity of the rearranged radical **7** (Figure 1), compared to the unrearranged radical **6**. The more nucleophilic rearranged radical therefore reacts selectively with the electrophile rather than with the silane, resulting in electrophile trapping rather than reduction.

**Figure 2:**
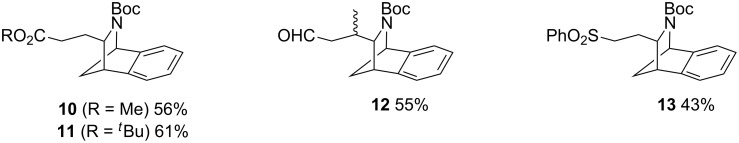
Other products obtained from xanthate **5** by tandem deoxygenation–rearrangement–electrophile trapping.

Encouraged by the above initial result, a number of other electrophiles were considered for the tandem deoxygenation–rearrangement–trapping reaction (Figure 2). Acrylate esters proved effective electrophiles: methyl acrylate and *tert*-butyl acrylate gave rearranged trapped products **10** and **11** in yields of 56% and 61%, respectively. α,β-Unsaturated aldehydes were also tolerated: crotonaldehyde gave a separable mixture (~2:1) of diastereomeric aldehydes **12** in a combined yield of 55%. Deoxygenation–rearrangement–electrophile trapping with acrolein was also considered, however the practical difficulties of using this reagent (prone to polymerisation in the absence of a radical inhibitor) precluded its application. Using 3-methyl-2-butenal led to only rearranged–reduced product **8** (79%). In many cases, radical reactions are considered to proceed through early transition states and, as such, steric effects are not usually significant [7]. Therefore, the lack of reactivity with 3-methyl-2-butenal could be due to the positive inductive effect of the terminal methyl groups rendering the β-position of the aldehyde less electrophilic.

In an attempt to access a simple trapped aldehyde indirectly, acrolein diethyl acetal was tested as a potential electrophile, however only the rearranged–reduced product **8** was obtained (81%); 1-heptene similarly gave only rearranged–reduced azacycle **8**, in 81% yield. These latter results were indicative of a generally-observed trend that alkenes bearing less strongly electron-withdrawing substituents were less effective electrophiles in this reaction. Phenyl vinyl sulfone gave trapped rearranged azacycle **13** in 43% yield, with rearranged–reduced azacycle **8** also isolated in 38% yield, suggesting that the rate of electrophile trapping with the sulfone corresponds approximately to the rate of hydrogen atom transfer from (TMS)_3_SiH. Attempted reaction with methyl vinyl ketone, *N*,*N*-dimethylacrylamide and methyl propiolate gave rearranged-reduced azacycle **8** in modest yields, as well as small quantities of hydrosilylated electrophile (for details see Supporting Information File 1) [26].

Having probed the generality of the reaction, attempts were made to optimise the yields of the electrophile trapping. These studies were performed with phenyl vinyl sulfone, since this had undergone partial electrophile trapping and partial rearrangement–reduction under the standard conditions. It was considered that a greater excess of the electrophile might bias the product distribution in favour of electrophile trapping; however, it was found that increasing the concentration of the olefin resulted in a decrease in the yield of the desired rearranged–trapped azacycle **13**. This result, in conjunction with the silylated by-products recovered from these reactions, supports the hypothesis that (reversible) hydrosilylation of the electrophile may be a significant competing pathway in these reactions.

**Scheme 3:**
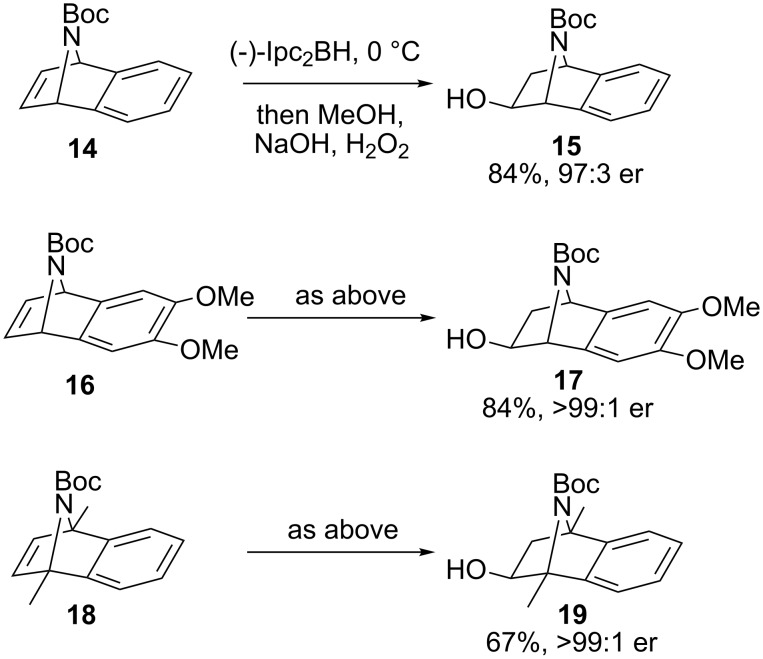
Asymmetric hydroboration–oxidation of alkenes **14**, **16** and **18**.

With a viable method for the deoxygenation-rearrangement-trapping of 2-azabenzonorbornenyl xanthates established, we next sought to achieve an asymmetric access to such systems by asymmetric hydroboration. Previous test reactions had indicated that efficient metal-catalysed asymmetric hydroboration [27] was difficult to achieve in this system [28] and therefore stoichiometric hydroboration was examined. The timely work of Laschat and co-workers in an analogous tropinone system [29] suggested the application of diisopinocampheylborane (Ipc_2_BH) [30–31], which with cycloadduct **14** at 0 °C gave alcohol **15** in 84% yield (68% at −10 °C, Scheme 3). HPLC analysis revealed that alcohol **15** formed in excellent enantiomeric ratio (er) [32] (97:3) at both temperatures.

Encouraged by the high hydroboration selectivity, two related cycloadducts, **16** [24] and **18** [28] with differing electronic and structural properties were examined (Scheme 3) which led to the corresponding alcohols **17** and **19** in similar ers.

**Scheme 4:**
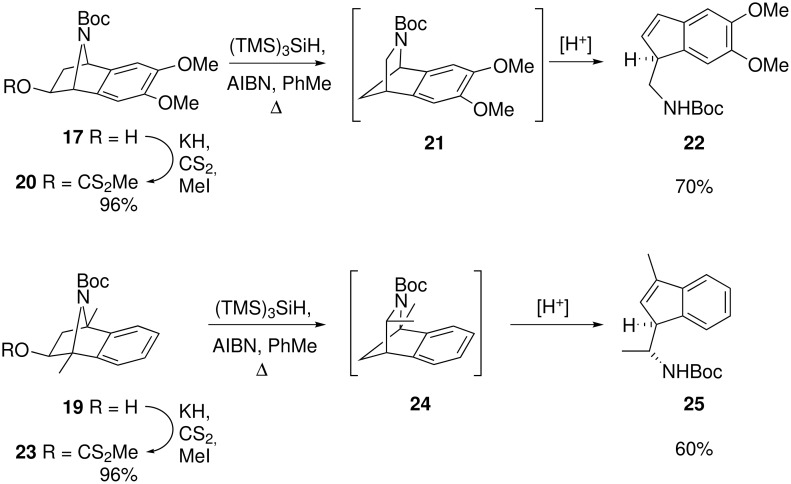
Deoxygenation–rearrangement–isomerisation of xanthates **20** and **23**.

With highly enantioenriched alcohols **17** and **19** in hand, the possibility of extending the tandem deoxygenation–rearrangement–electrophile trapping to these substrates was examined. Simple deoxygenation–rearrangement–reduction of the methyl xanthate derivatives **20** and **23** were performed as initial test reactions (Scheme 4). However, product profiles of these reactions were found to be inconsistent: the expected rearranged bridged azacycles **21** and **24** could be observed by ^1^H NMR, provided that the spectra were recorded immediately after isolation. But in most experiments some degree of isomerisation to (aminomethyl)indenes **22** and **25** respectively were observed, as indicated by the appearance of vinylic CH resonances in the ^1^H NMR spectra (δ 6.8–6.0). This isomerisation is presumably the result of trace acid catalysis, with the process likely proceeding via protonation of the nitrogen atom, followed by ring opening to a stabilised benzylic cation. ^1^H NMR analysis also revealed that the resulting (aminomethyl)indenes **22** and **25** degraded with time.

**Scheme 5:**
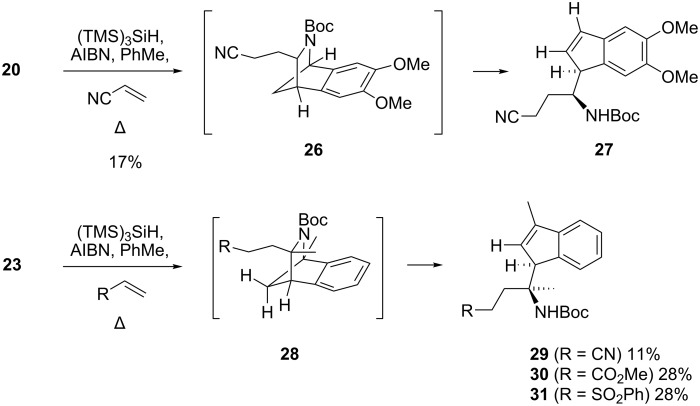
Deoxygenation–rearrangement–electrophile trapping of xanthates **20** and **23**.

The tandem deoxygenation–rearrangement–electrophile trapping reaction was attempted for xanthates **20** and **23** using acrylonitrile and, whilst the expected rearrangement–trapping occurred (as judged by diagnostic features in the crude ^1^H NMR spectra, for details see Supporting Information File 1), isomerisation of the initial adducts to the corresponding chromatographically sensitive indenes commenced rapidly; this may account for the modest isolated yields of the trapped azacycles (Scheme 5). Further electrophile trapping experiments were undertaken with dimethyl xanthate **23**: methyl acrylate and phenyl vinyl sulfone gave the corresponding indenes **30** and **31**, which could both be isolated, albeit in modest yields.

**Scheme 6:**
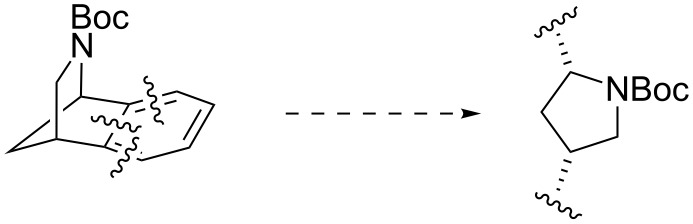
2-Azabenzonorbornene as a masked pyrrolidine.

Having established a stereoselective route to 3-*exo*-substituted 2-azabenzonorbornenes, further synthetic transformations of these adducts were addressed. Firstly, we considered that the 2-azabenzonorbornene framework could be a masked pyrrolidine: cleavage of the aromatic ring would reveal a stereodefined 2,4-disubstituted pyrrolidine (Scheme 6). This transformation was of interest because the pyrrolidine ring is a common nitrogen-containing motif in natural products and pharmaceutically relevant molecules [33].

**Scheme 7:**
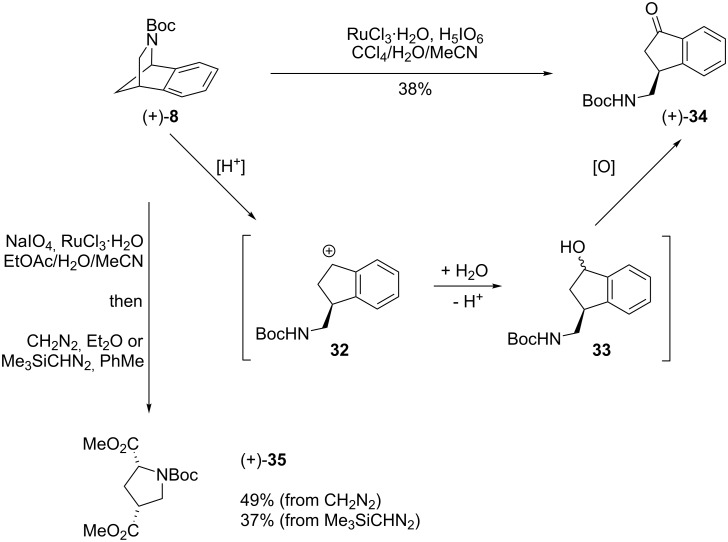
Ring-opening–hydration–oxidation of azacycle **8**.

Ruthenium tetraoxide was first examined as a reagent for the oxidative cleavage of the aromatic ring, since treatment of alkyl-substituted arenes is known to result in complete oxidation to the corresponding alkyl carboxylic acids [34–35]. In the event, application of standard literature oxidation conditions (RuCl_3_·H_2_O, H_5_IO_6_ in CCl_4_/H_2_O/MeCN) with rearranged–reduced azacycle **8** followed by attempted esterification (Me_3_SiCHN_2_) to aid isolation led only to a Boc-protected (aminomethyl)indanone (+)-**34** (38%, Scheme 7). Ring-opening may have been catalysed by the periodic acid followed by trapping of the resulting benzylic cation **32** by water to give alcohol **33** and subsequent oxidation by RuO_4_.

**Scheme 8:**
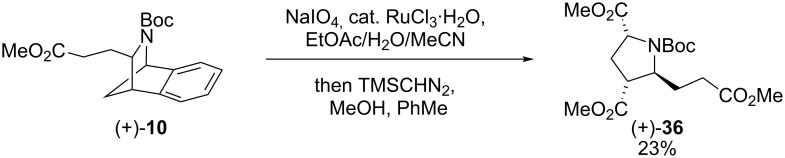
Preparation of trisubstituted pyrrolidine (+)-**36**.

Switching to sodium periodate as the stoichiometric oxidant and esterification using CH_2_N_2_ gave diester **35** (49%, 37% using Me_3_SiCHN_2_). Attempts to improve the procedure further by the use of alternative solvents proved unsatisfactory. Azacycle (+)-**10**, the product of deoxygenation-rearrangement-trapping with methyl acrylate (Figure 2), was subject to the same conditions as azacycle (+)-**8** and was found to give trisubstituted pyrrolidine **36**, in 23% yield (Scheme 8).

**Scheme 9:**
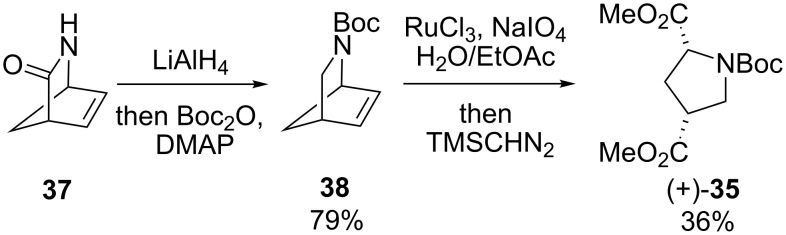
Preparation of pyrrolidine diester (+)-**35** from Vince’s lactam **37**.

Although the yields for these reactions were modest, access to pyrrolidine (+)-**35** allowed the absolute sense of asymmetric induction in the hydroboration–oxidation with (−)-Ipc_2_BH to be determined by chemical correlation. Pyrrolidine **35** was independently prepared from (1*R*)-(−)-2-azabicyclo[2.2.1]hept-5-en-3-one (Vince’s lactam [36], **37**) via reduction with LiAlH_4_, Boc-protection and oxidative cleavage [37] (Scheme 9). The pyrrolidines prepared from rearranged azacycle (+)-**8** and lactam **37** were both dextrorotatory, confirming the provisional assignment of configuration that had been made by analogy with Laschat’s work [29].

**Scheme 10:**
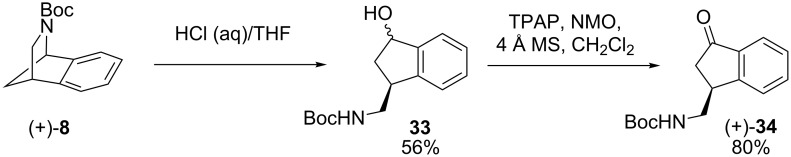
Acid-catalysed ring-opening–oxidation of azacycle (+)-**8**.

Since attempts to effect RuO_4_-mediated oxidative cleavage of 2-azabenzonorbornene **8** employing periodic acid as the stoichiometric co-oxidant resulted in the formation of (aminomethyl)indanone **34**, the possibility of performing this reaction with a milder oxidant was investigated. If the putative mechanism for the reaction was correct (Scheme 7), it seemed likely that a similarly strong acid would be needed. After some test reactions, the use of a 1:1 mixture of aqueous HCl and THF was found to consume azacycle (+)-**8** to give (aminomethyl)indanol **33** (56% yield) and subsequent oxidation using NMO/TPAP [38] gave (aminomethyl)indanone (+)-**34** (80%, Scheme 10).

**Scheme 11:**
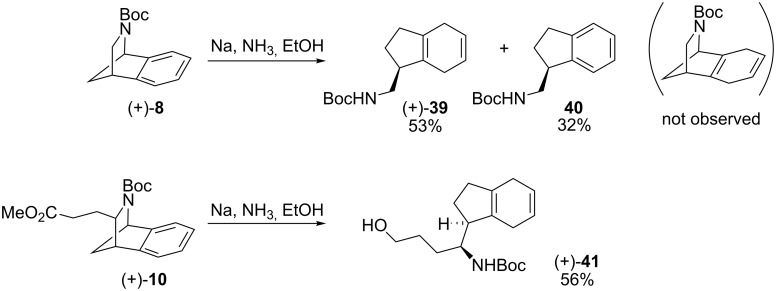
Birch reduction of (+)-**8** and **10**.

Having examined oxidative cleavage of the aromatic portion of the 2-azabenzonorbornenyl framework and acid-catalysed rearrangement, reduction also presented an attractive means of modifying the carbon skeleton. Reduction of azacycle (+)-**8** under standard Birch conditions [39], gave diene (+)-**39** (53% yield, Scheme 11) and indane **40** (32%). Birch reduction of substituted 2-azabenzonorbornene (+)-**10** gave diene (+)-**41** (56%), where concomitant reduction of the ester functionality had occurred.

## Conclusion

Electrophile trapping in stannane-mediated dehalogenations and silane-mediated xanthate deoxygenations have previously been reported [1,14]. The present work provides examples in which the intermediate radical undergoes rearrangement prior to electrophile trapping, thereby providing a new route to substituted 2-aza-5,6-benzonorbornenes. These adducts have been shown to lead on to pharmaceutically significant [40] (aminomethyl)indenes, and to substituted pyrrolidines and tetrahydroindenes following oxidative and reductive transformations, respectively.

## Supporting Information

File 1 Full preparative details of all compounds prepared are reported, together with their spectroscopic data.

## References

[1] Giese B (1986). Radicals in Organic Synthesis: Formation of Carbon-Carbon Bonds.

[2] Motherwell W B, Crich D (1992). Free Radical Chain Reactions in Organic Synthesis.

[3] Renaud P, Sibi M P (2001). Radicals in Organic Synthesis.

[4] Togo H (2004). Advanced Free Radical Reactions for Organic Synthesis.

[5] Gansäuer A (2006). Radicals in Synthesis I.

[6] Gansäuer A (2006). Radicals in Synthesis II.

[7] Jasperse C P, Curran D P, Fevig T L (1991). Chem Rev.

[8] Curran D P (1988). Synthesis.

[9] Curran D P (1988). Synthesis.

[10] Curran D P (1991). Synlett.

[11] Bowry V, Lusztyk J, Ingold K U (1990). Pure Appl Chem.

[12] Cristol S J, Brindell G D, Reeder J A (1958). J Am Chem Soc.

[13] Cristol S J, Davies D I (1964). J Org Chem.

[14] Hodgson D M, Winning L H (2007). Org Biomol Chem.

[15] Hodgson D M, Maxwell C R, Matthews I R (1998). Synlett.

[16] Hodgson D M, Maxwell C R, Wisedale R, Matthews I R, Carpenter K J, Dickenson A H, Wonnacott S (2001). J Chem Soc, Perkin Trans 1.

[17] Hodgson D M, Hachisu S, Andrews M D (2005). Org Lett.

[18] Hodgson D M, Hachisu S, Andrews M D (2005). J Org Chem.

[19] Hodgson D M, Galano J-M (2005). Org Lett.

[20] Studer A, Bossart M (2001). Tetrahedron.

[21] Hodgson D M, Winning L H (2006). Synlett.

[22] Ballestri M, Chatgilialoglu C, Clark K B, Griller D, Giese B, Kopping B (1991). J Org Chem.

[23] Chatgilialoglu C (2004). Organosilanes in Radical Chemistry.

[24] Hodgson D M, Bebbington M W P, Willis P (2003). Org Biomol Chem.

[25] Hodgson D M, Bebbington M W P, Willis P (2002). Org Lett.

[26] Kopping B, Chatgilialoglu C, Zehnder M, Giese B (1992). J Org Chem.

[27] Beletskaya I, Pelter A (1997). Tetrahedron.

[R28] 28Bebbington, M. W. P. Nitrogen-directed free radical rearrangements. D.Phil. Thesis, University of Oxford, U.K., 2002.

[29] Cramer N, Laschat S, Baro A, Frey W (2003). Synlett.

[30] Brown H C, Ramachandran P V (1995). J Organomet Chem.

[31] Brown H C, Singram B (1984). J Org Chem.

[32] Gawley R E (2006). J Org Chem.

[33] O’Hagan D (2000). Nat Prod Rep.

[34] Mander L N, Williams C M (2003). Tetrahedron.

[35] Piatak D M, Herbst G, Wicha J, Caspi E (1969). J Org Chem.

[36] Daluge S, Vince R (1978). J Org Chem.

[37] Arakawa Y, Yasuda M, Ohnishi M, Yoshifuji S (1997). Chem Pharm Bull.

[38] Ley S V, Norman J, Griffith W P, Marsden S P (1994). Synthesis.

[39] Birch A J (1944). J Chem Soc.

[40] Trivedi B K (1989). Antihyperlipidemic and Antiatherosclerotic Compounds and Compositions. Eur. Pat. Appl..

