# Supplementary material for: Radical cascades using enantioenriched 7-azabenzonorbornenes and their applications in synthesis
Source: Beilstein J Org Chem. 2008 Oct 24;4:38. doi: 10.3762/bjoc.4.38 (PMC2587947; doi:10.3762/bjoc.4.38)
Supplement: File 1 — Full preparative details of all compounds prepared are reported, together with their spectroscopic data. [file Beilstein_J_Org_Chem-04-38-s001.doc]

**Radical cascades using enantioenriched 7-azabenzonorbornenes and their applications in synthesis**

David M. Hodgson* and Leonard H. Winning

*Address: Department of Chemistry, Chemistry Research Laboratory, University of Oxford, Mansfield Road, Oxford, OX1 3TA, UK; Fax +44(1865) 285002*

Email: David M. Hodgson* - david.hodgson@chem.ox.ac.uk

* Corresponding author

***Supporting Information File 1***

Table of contents

4 General remarks

5 *tert*-Butyl (1*S*,8*R*,10*S*)-10-(2-cyanoethyl)-9-azatricyclo[6.2.1.02,7]undeca-2,4,6-triene-9-carboxylate (**9**)

6 *tert*-Butyl (1*S*,8*R*,10*S*)-10-(3-methoxy-3-oxopropyl)-9-azatricyclo[6.2.1.02,7]undeca-2,4,6-triene-9-carboxylate(**10**)

7 *tert*-Butyl (1*RS*,8*SR*,10*RS*)-10-(3-*tert*-butoxy-3-oxopropyl)-9-azatricyclo[6.2.1.02,7]undeca-2,4,6-triene-9-carboxylate(**11**)

8 *tert*-Butyl (1*RS*,8*SR*,10*RS*)-10-(1-oxobut-3-yl)-9-azatricyclo[6.2.1.02,7]undeca-2,4,6-triene-9-carboxylate(**12**)

9 *tert*-Butyl (1*S*,8*R*,10*S*)-10-[2-(phenylsulfonyl)ethyl]-9-azatricyclo[6.2.1.02,7]undeca-2,4,6-triene-9-carboxylate(**13**)

10 1-[tris(Trimethylsilyl)silyl]butan-3-one

10 *N*,*N*-Dimethyl-3-[tris(trimethylsilyl)silyl]propanamide

11 *tert*-Butyl (1*R*,8*R*,9*S*)-9-hydroxy-11-azatricyclo[6.2.1.02,7]undeca-2,4,6-triene-11-carboxylate(**15**)

11 *tert*-Butyl (1*R*,8*R*,9*S*)-9-hydroxy-4,5-dimethoxy-11-azatricyclo[6.2.1.02,7]undeca-2,4,6-triene-11-carboxylate(**17**)

12 *tert*-Butyl (1*R*,8*R*)-4,5-dimethoxy-9-oxo-11-azatricyclo[6.2.1.02,7]undeca-2,4,6-triene-11-carboxylate

13 *tert*-Butyl (1*R*,8*R*,9*S*)-9-hydroxy-1,8-dimethyl-11-azatricyclo[6.2.1.02,7]undeca-2,4,6-triene-11-carboxylate(**19**)

14 *tert*-Butyl (1*R*,8*R*,9*S*)-9-[(methylsulfanyl)thiocarbonyloxy]-4,5-dimethoxy-11-azatricyclo[6.2.1.02,7]undeca-2,4,6-triene-11-carboxylate(**20**)

14 *tert*-Butyl (1*R*,8*S*)-4,5-dimethoxy-9-azatricyclo[6.2.1.02,7]undeca-2,4,6-triene-9-carboxylate(**21**)

15 *tert*-Butyl (1*R*,8*R*,9*S*)-9-[(methylsulfanyl)thiocarbonyloxy]-1,8-dimethyl-11-azatricyclo[6.2.1.02,7]undeca-2,4,6-triene-11-carboxylate (**23**)

16 *tert*-Butyl {(*R*)-1-[(*S*)-3-methyl-1*H*-inden-1-yl]ethyl}carbamate(**25**)

16 *tert*-Butyl {(*S*)-3-cyano-1-[(*S*)-5,6-dimethoxy-1*H*-inden-1-yl]propyl}carbamate(**27**)

17 *tert*-Butyl {(*S*)-4-cyano-2-[(*S*)-3-methyl-1*H*-inden-1-yl]butan-2-yl}carbamate(**29**)

17 Methyl (*S*)-4-(*tert*-butoxycarbonylamino)-4-[(*S*)-3-methyl-1*H*-inden-1-yl]pentanoate(**30**)

18 *tert*-Butyl {(*S*)-2-[(*S*)-3-methyl-1*H*-inden-1-yl]-4-(phenylsulfonyl)butan-2-yl}carbamate (**31**)

19 *tert*-Butyl (1*S*,8*R*)-9-azatricyclo[6.2.1.02,7]undeca-2,4,6-triene-9-carboxylate(**8**)

201-*tert*-Butyl 2,4-dimethyl (2*R*,4*R*)-pyrrolidine-1,2,4-tricarboxylate(**35**)

21 1-*tert*-Butyl 2,4-dimethyl (2*R*,4*R*,5*S*)-5-(3-methoxy-3-oxopropyl)pyrrolidine-1,2,4-tricarboxylate(**36**)

21 *tert*-Butyl (1*R*,4*S*)-2-azabicyclo[2.2.1]hept-5-ene-2-carboxylate(**38**)

221-*tert*-Butyl 2,4-dimethyl (2*R*,4*R*)-pyrrolidine-1,2,4-tricarboxylate(**35**)

22 *tert*-Butyl [(3-hydroxy-2,3-dihydro-1*H*-inden-1-yl)methyl]carbamate (mixture of diastereomers) (**33**)

23 *tert*-Butyl [(*S*)-(3-oxo-2,3-dihydro-1*H*-inden-1-yl)methyl]carbamate(**34**)

24 *tert*-Butyl {[(*S*)-2,3,4,7-tetrahydro-1*H*-inden-1-yl]methyl}carbamate (**39**)

25 *tert*-Butyl {(*S*)-4-hydroxy-1-[(*S*)-2,3,4,7-tetrahydro-1*H*-inden-1-yl]butyl}carbamate(**41**)

26 References

**General Remarks**

All reactions requiring anhydrous conditions were performed in oven-dried or flame-dried glassware under an inert atmosphere (argon or nitrogen). CH2Cl2, Et2O, THF, PhMe and MeCN were degassed and dried over alumina according to the procedure of Grubbs and co-workers [1]. All other reagents were used as received unless otherwise stated. Reactions were monitored by TLC using Merck aluminium-backed plates pre-coated with silica (0.25 mm, 60, F254). The plates were visualised under UV light and developed using solutions of phosphomolybdic acid, vanillin or basic KMnO4. Removal of solvent under reduced pressure was performed using Büchi rotary evaporators, achieving a minimum pressure of ca. 15 mbar, followed by drying at 0.1 mbar using an oil pump. Column chromatography was performed on silica [Kieselgel 60 (40–63 μm)]. Petroleum ether refers to the fraction boiling in the range 30–40 °C. Melting points were determined using a Griffin Melting Point Apparatus. Optical rotations were measured using a Perkin-Elmer 241 Polarimeter with a cell of path length 10.0 cm; concentrations are quoted in g/100 mL; specific rotations are given in 10-1 deg cm2 g-1. Infrared spectra were recorded on a Perkin-Elmer 1750 FTIR or a Bruker Tenso 27 FTIR spectrometer; absorptions are quoted in wavenumbers (cm-1) and are classified as s (strong), m (medium), w (weak) and/or br (broad); only selected absorptions are recorded. 1H and 13C NMR spectra were recorded on Bruker DPX 200, DPX 250, DPX 400, DQX 400 or AMX 500 spectrometers; chemical shifts (δ) are quoted in parts per million, referenced to the residual solvent peak as an internal standard [2]; coupling constants (*J*) are quoted in Hz. High-resolution mass spectra were obtained by chemical ionisation (NH3 and Na+) or by GC analysis with a BPX5 column-HP 6890 (dimethyl silicone capillary column, *l* = 30 m, *φ* = 0.25 mm) equipped with a reflectron TOF mass spectrometer (60 eV, He flow rate = 1 mL min-1). Chiral GC analyses were carried out using a CE Instruments Trace GC (Thermoquest) chromatograph, fitted with an SGE Cydex-B column. Chiral HPLC analyses were carried out using Daicel Chiracel OD, AD or OJ columns (*l* = 250 mm, diameter = 4.6 mm) on a Gilson System with 712 controller software and 188 UV/vis detector, operating at 255 or 224 nm.

***tert*-Butyl (1*S*,8*R*,10*S*)-10-(2-cyanoethyl)-9-azatricyclo[6.2.1.02,7]undeca-2,4,6-triene-9-carboxylate (9)**

Xanthate (+)-**5** [3] (200 mg, 0.57 mmol) was dissolved in PhMe (20 mL) and the solution was heated to reflux. (TMS)3SiH (0.26 mL, 0.85 mmol), AIBN (48 mg, 0.29 mmol) and acrylonitrile (44 mg, 0.83 mmol) were dissolved in PhMe (4 mL) and added *via* syringe pump over 100 min. The reaction was refluxed for a further 30 min, then cooled and the solvent removed under reduced pressure. Column chromatography (gradient elution, 0–60% Et2O in petroleum ether) gave nitrile **9** as a yellow oil (131 mg, 77%). *R*f (1:1 Et2O : petroleum ether) 0.29; [α]25D +73.4 (CHCl3, *c* = 1.00); νmax (thin film) 2978m, 3348w (CN), 1694s (CO), 1462m, 1367s, 1249m, 1177s, 1142s, 1122m, 1101w, 1074m, 999w, 913w, 840w, 758m, 734s; H (CDCl3, 400MHz) 7.44–7.08 (4H, m, 4 × aromatic CH), 4.99 and 4.89 (1H, s and s, rotamers, NCH), 3.41 (1H, s, CH), 3.07–2.94 (1H, m, C*H*CH2CH2), 2.65–2.43 (2H, m, CH2CN), 2.28–2.03 (2H, m, C*H*H and C*H*HCH2CN), 2.02–1.85 (1H, m, CH*H*CH2CN), 1.80 (1H, d, *J* = 9, CH*H*), 1.41 (9H, s, C(CH3)3); C (CDCl3, 100 MHz) 156.9 (CO), 145.2 (quat aromatic), 144.3 (quat aromatic), 127.2 (aromatic CH), 126.2 (aromatic CH), 121.7 (aromatic CH), 121.3 and 120.0 (rotamers, CN), 120.6 (aromatic CH), 80.0 (*C*(CH3)3), 62.9 (NCH), 59.5 (N*C*HCH2CH2CN), 48.2 (CH), 45.4 (CH2), 31.9 (*C*H2CH2CN), 28.4 (C(*C*H3)3), 25.5 (*C*H2CN); *m/z* (CI+) 299 (MH+ 10%), 260 (40), 243 (25), 199 (100), 117 (40); *m/z* (CI+) C18H23N2O2 (MH+) requires 299.1760, found 299.1768. Assignment was made by NOE analysis of **9**, compared to a previous corresponding analysis of structurally related azatricycle **A**: when **9** was irradiated at the resonant frequency of C11-H, the side-chain methylene group indicated below showed an enhancement, whereas the C10-H proton did not.

***tert*-Butyl (1*S*,8*R*,10*S*)-10-(3-methoxy-3-oxopropyl)-9-azatricyclo[6.2.1.02,7]undeca-2,4,6-triene-9-carboxylate (10)**

Xanthate (+)-**5** [3](250 mg, 0.71 mmol) was dissolved in PhMe (20 mL) and the solution was heated to reflux. (TMS)3SiH (0.33 mL, 1.1 mmol), AIBN (58 mg, 0.36 mmol) and methyl acrylate (90 mg, 1.1 mmol) were dissolved in PhMe (4 mL) and added *via* syringe pump over 100 min. The reaction was refluxed for a further 30 min, then cooled and the solvent removed under reduced pressure. Column chromatography (gradient elution, 5–20% Et2O in petroleum ether) gave ester **10** as a colourless oil (132 mg, 56%). *R*f (1:1 Et2O : petroleum ether) 0.75; [α]25D +81.0 (CHCl3, *c* = 1.00); νmax (thin film) 2977m, 1739s (OC=O), 1695s (NC=O), 1462m, 1366s, 1260m, 1170s, 1121m, 1100m, 1074m, 1000w, 910w, 839w, 757m; H (CDCl3, 400 MHz) 7.39–7.05 (4H, m, 4 × aromatic CH), 4.98 and 4.86 (0.25H and 0.75H, s and s, rotamers, NCH), 3.67 (3H, s, OCH3), 3.33 (1H, s, CH), 3.04–2.92 and 2.92–2.79 (0.75H and 0.25H, m and m, rotamers, NCH), 2.59–2.44 (2H, m, CH2), 2.30–2.08 (2H, m, CH2), 1.98–1.79 (1H, m, C*H*H), 1.79–1.71 (1H, m, CH*H*), 1.43–1.35 and 1.35–1.26 (9H, m, C(CH3)3); C (CDCl3, 100 MHz) 173.9 and 173.6 (rotamers, CO2Me), 156.6 (CO2*t*Bu), 145.7 (quat. aromatic), 144.2 (quat. aromatic), 126.9 (aromatic CH), 126.3 and 125.9 (rotamers, aromatic CH), 121.5 and 121.1 (rotamers, aromatic CH), 120.4 (aromatic CH), 79.5 (*C*(CH3)3), 62.7 and 61.7 (rotamers, CH), 59.8 (CH), 51.8 and 51.5 (rotamers, OCH3), 48.2 and 47.8 (rotamers, CH), 45.3 and 44.8 (rotamers, CH2), 32.6 and 31.9 (CH2), 30.5 and 30.3 (rotamers, CH2), 28.5 and 28.3 (rotamers, C(*C*H3)3); *m/z* (CI+) 332 (MH+ 100%), 276 (43), 232 (77), 214 (12), 200 (5), 183 (4), 172 (10), 158 (15), 144 (17), 130 (11), 116 (26); *m/z* (CI+) C19H26NO4 (MH+) requires 332.1865, found 332.1866.

***tert*-Butyl (1*RS*,8*SR*,10*RS*)-10-(3-*tert*-butoxy-3-oxopropyl)-9-azatricyclo[6.2.1.02,7]undeca-2,4,6-triene-9-carboxylate (11)**

Xanthate **5** [4] (100 mg, 0.28 mmol) was dissolved in PhMe (8 mL) and the solution was heated to reflux. (TMS)3SiH (0.13 mL, 0.44 mmol), AIBN (24 mg, 0.14 mmol) and *tert*-butyl acrylate (53 mg, 0.43 mmol) were dissolved in PhMe (2 mL) and added *via* syringe pump over 100 min. The reaction was refluxed for a further 30 min, then cooled and the solvent removed under reduced pressure. Column chromatography (gradient elution, 0–60% Et2O in petroleum ether) gave ester **11** as a yellow oil (66 mg, 61%). *R*f (1:1 Et2O : petroleum ether) 0.31; max (thin film) 3433w, 2977m, 2931m, 1729s, 1701s, 1458w, 1392m, 1307s, 1256m, 1152s, 1072w, 842m; H (CDCl3, 400 MHz) 7.29–7.06 (4H, m, 4 × aromatic CH), 4.99 and 4.87 (0.3H and 0.7H, s and s, rotamers, NCH), 3.42–3.31 (1H, m, CH), 3.04–2.91 and 2.91–2.80 (0.7H and 0.3H, m and m, rotamers, NCH), 2.41–2.34 (2H, m, CH2), 2.25–2.09 (2H, m, CH2), 1.93–1.80 (1H, m, C*H*H), 1.80–1.73 (1H, m, CH*H*), 1.42 and 1.45 (9H, s and s, rotamers, C(CH3)3), 1.42 and 1.40 (9H, s and s, rotamers, C(CH3)3); δC (CDCl3, 100 MHz) [*C*O*C*(CH3)3 × 2 not observed] 145.2 (quat. aromatic), 144.2 (quat. aromatic), 127.1 and 127.0 (rotamers, aromatic CH), 126.1 (aromatic CH), 121.6 and 121.4 (rotamers, aromatic CH), 120.2 (aromatic CH), 61.9 (CH), 60.7 (CH), 48.6 and 48.4 (rotamers, CH), 43.9 (CH2), 41.2 (CH2), 39.4 (CH2), 28.6 and 28.4 (carbamate C(*C*H3)3), 23.6 and 23.4 (ester C(*C*H3)3); *m/z* (CI+) 374 (MH+, 100%), 318 (30), 282 (15), 264 (10), 215 (25); *m/z* (CI+) C22H32NO4 (MH+) requires 374.2326, found 374.2326.

***tert*-Butyl (1*RS*,8*SR*,10*RS*)-10-(1-oxobut-3-yl)-9-azatricyclo[6.2.1.02,7]undeca-2,4,6-triene-9-carboxylate (12)**

Xanthate **5** [4] (100 mg, 0.28 mmol) was dissolved in PhMe (8 mL) and the solution was heated to reflux. (TMS)3SiH (0.13 mL, 0.44 mmol), AIBN (24 mg, 0.14 mmol) and crotonaldehyde (0.04 mL, 0.43 mmol) were dissolved in PhMe (1 mL) and added *via* syringe pump over 100 min. The reaction was refluxed for a further 30 min, then cooled and the solvent removed under reduced pressure. Column chromatography (gradient elution, 2–40% Et2O in pentane) gave two diastereomers of azacycle **12**. First to elute was a yellow oil (33 mg, 37%): *R*f (80% Et2O : pentane) 0.36; νmax (thin film) 3443w, 2950s, 2893m, 1707s, 1682s, 1458m, 1367s, 1295m, 1244s, 1212w, 1162s, 1108s, 1084m; δH (400 MHz, CDCl3) 9.80 (1H, s, CHO), 7.38–7.07 (4H, m, 4 × aromatic CH), 5.12 and 4.95 (0.4H and 0.6H, rotamers, NCH), 3.62 (1H, s, CH), 3.56–3.50 (1H, m, CH *endo*), 2.97–2.90 (1H, m, C*H*HCHO), 2.42–2.25 (1H, m, C*H*CH3), 2.22 (1H, d, *J* = 9, C*H*H), 2.05–1.92 (1H, m, C*H*CHO), 1.88 (1H, d, *J* = 9, CH*H*), 1.44 (9H, s, C(CH3)3), 1.38 (3H, d, *J* = 7, CH3); δC (CDCl3, 100 MHz) 202.7 (CHO), 155.5 and 154.8 (rotamers, CO), 145.5 and 145.3 (rotamers, quat. aromatic), 144.3 and 144.2 (rotamers, quat. aromatic), 127.0 and 126.9 (rotamers, aromatic CH), 126.1 and 125.8 (rotamers, aromatic CH), 121.6 and 121.4 (rotamers, aromatic CH), 120.9 and 121.4 (rotamers, aromatic CH), 79.8 and 79.3 (rotamers, *C*(CH3)3), 63.2 and 61.8 (rotamers, NCH), 48.8 and 48.6 (rotamers, CH2), 48.5 and 48.4 (rotamers, CH2) 46.8 (CH), 45.4 and 44.5 (rotamers, NCH), 30.88 (CH), 28.5 and 28.4 (rotamers, C(*C*H3)3), 28.2 (CH3); *m*/*z* (CI+) 316 (14%), 298 (5), 274 (9), 260 (32), 242 (11), 216 (57), 198 (100), 187 (3), 170 (17), 144 (41), 116 (13), 100 (5), 72 (8), 57 (2); *m*/*z* (CI+) C19H26NO3 (MH+) requires 316.1913, found 316.1911. Second to elute was a yellow oil (16 mg, 18%): *R*f (4:1 Et2O : pentane) 0.25; νmax (thin film) 2977m, 2932m, 2887w, 1694s, 1478m, 1461m, 1391s, 1366s, 1310w, 1295w, 1275m, 1251m, 1180s, 1153s, 1092m, 1070m, 1015w; δH (400 MHz, CDCl3) 9.80 (1H, t, *J* = 2, CHO), 7.39–7.07 (4H, m, 4 × aromatic CH), 5.02 and 4.89 (1H, rotamers, NCH), 3.42 (1H, s, CH), 2.94–2.80 (1H, m, NCH), 2.75–2.64 (1H, m, C*H*HCHO), 2.60–2.43 (1H, m, C*H*CH3), 2.44–2.29 (1H, m, CH*H*CHO), 2.15 (1H, d, *J* = 9, C*H*H), 1.72 (1H, d, *J* = 9, CH*H*), 1.41 (9H, s, C(CH3)3), 1.14 (3H, d, *J* = 7, CH3); δC (CDCl3, 100 MHz) [*C*(CH3)3 not observed] 206.9 (CHO), 158.4 (C=O), 146.2 (quat. aromatic), 144.7 (quat. aromatic), 127.0 (aromatic CH), 125.9 (aromatic CH), 121.4 (aromatic CH), 120.6 (aromatic CH), 64.6 (NCH), 62.9 (NCH), 49.0 (CH2), 46.5 (CH), 45.4 (CH2), 32.6 (CH), 28.4 (C(*C*H3)3), 18.1 (CH3); *m*/*z* (CI+, NH3) 316 (MH+ 55%), 279 (17), 246 (11), 260 (30), 216 (95), 206 (30), 198 (100), 72 (5), 51 (7); *m*/*z* (CI+, NH3) C19H26NO3 (MH+) requires 316.1913, found 316.1912.

***tert*-Butyl (1*S*,8*R*,10*S*)-10-[2-(phenylsulfonyl)ethyl]-9-azatricyclo[6.2.1.02,7]undeca-2,4,6-triene-9-carboxylate (13)**

Xanthate (+)-**5** [4] (250 mg, 0.71 mmol) was dissolved in PhMe (20 mL) and the solution was heated to reflux. (TMS)3SiH (0.33 mL, 1.1 mmol), AIBN (60 mg, 0.36 mmol) and phenyl vinyl sulfone (177 mg, 1.1 mmol) were dissolved in PhMe (5 mL) and added *via* syringe pump over 100 min. The reaction was refluxed for a further 30 min, then cooled and the solvent removed under reduced pressure. Column chromatography (gradient elution, 5–40% Et2O in petroleum ether) gave two products. First to elute was rearranged-reduced azacycle **8** [4]as a white solid (66 mg, 38%). Second to elute was sulfone **13** as a colourless oil (127 mg, 43%). *R*f (1:4 Et2O : petroleum ether) 0.09; [α]25D +56.9 (CHCl3, *c* = 1.00); νmax (thin film) 2923s, 2853s, 1691m, 1461s, 1377s, 1307m, 1247w, 1149w, 1086w; H (250 MHz, DMSO-*d*6, 90 °C) 7.97–7.92 (2H, m, 2 × phenyl CH), 7.82–7.61 (3H, m, 3 × phenyl CH), 7.36–7.06 (4H, m, 4 × aromatic CH), 4.83 (1H, s, NCH), 3.61–3.35 (2H, m, CH2SO2), 3.04 (1H, s, NCH), 2.91–2.83 (1H, m, CH), 2.25–2.12 (1H, m, C*H*HCH2SO2), 2.04 (1H, d, *J =* 9, C*H*H), 1.97–1.80 (1H, m, CH*H*CH2SO2), 1.67 (1H, d, *J* = 9, CH*H*), 1.30 (9H, s, C(CH3)3); C (CDCl3, 100 MHz) 156.9 (C=O), 145.2 (fused quat. aromatic), 144.3 (fused quat. aromatic), 139.2 (phenyl quat. aromatic), 133.7 (phenyl CH), 129.3 (2 × phenyl CH), 128.0 (2 × phenyl CH), 127.1 (aromatic CH), 126.1 (aromatic CH), 121.6 (aromatic CH), 120.6 (aromatic CH), 79.9 (*C*(CH3)3), 63.1 (*C*HNCH), 59.0 (*C*HNCH2­), 54.6 (CH), 48.6 (*C*H2­SO2), 45.5 (CH2), 29.1 (*C*H2CH2SO2), 28.4 (C(*C*H3)3); *m*/*z* (EI+) 413 (M+, 10%), 340 (20), 312 (100), 216 (20); *m*/*z* (EI+) C23H27O4NS (M+) requires 413.1655, found 413.1654.

**1-[tris(Trimethylsilyl)silyl]butan-3-one**

Xanthate **5** [4] (250 mg, 0.71 mmol) was dissolved in PhMe (20 mL) and the solution was heated to reflux. (TMS)3SiH (0.09 mL, 1.1 mmol), AIBN (60 mg, 0.36 mmol) and methyl vinyl ketone (77 mg, 1.1 mmol) were dissolved in PhMe (5 mL) and added *via* syringe pump over 100 min. The reaction was refluxed for a further 30 min, then cooled and the solvent removed under reduced pressure. Column chromatography (gradient elution, 5–60% Et2O in petroleum ether) gave rearranged-reduced azacycle **8** [4] as a yellow oil (58 mg, 38%) and the title silane [5]as a yellow oil (21 mg, 6%). *R*f (1:4 Et2O : petroleum ether) 0.58; νmax (thin film) 2950s, 2894m, 1720s, 1409w, 1357w, 1245s, 1189w, 836s, 747w, 687m, 623m; δH (CDCl3, 400 MHz) 2.48–2.41 (2H, m, CH2), 2.15 (3H, s, CH3), 1.06–0.88 (2H, m, CH2), 0.18 (27H, s, Si[Si(CH3­)3]3); δC (CDCl3, 100 MHz) 209.4 (CO), 42.7 (CH2), 29.1 (CH3), 1.3 (CH2), 1.0 (Si[Si(CH3­)3]3).

***N*,*N*-Dimethyl-3-[tris(trimethylsilyl)silyl]propanamide**

Xanthate **5** [4](250 mg, 0.71 mmol) was dissolved in PhMe (20 mL) and the solution was heated to reflux. (TMS)3SiH (0.09 mL, 1.1 mmol), AIBN (60 mg, 0.36 mmol) and *N*,*N*-dimethylacrylamide (109 mg, 1.1 mmol) were dissolved in PhMe (5 mL) and added *via* syringe pump over 100 min. The reaction was refluxed for a further 30 min, then cooled and the solvent removed under reduced pressure. Column chromatography (gradient elution, 5–60% Et2O in petroleum ether) gave rearranged-reduced azacycle **8** [4]as a yellow oil (56 mg, 37%) and the title silane as a yellow oil (81 mg, 9%). *R*f (1:4 Et2O : petroleum ether) 0.07; νmax (thin film) 2948s, 2894m, 1653s, 1395m, 1245s, 1126w, 835s, 733w, 688w, 622m; δH (CDCl3, 400 MHz) 2.97 (3H, s, CH3), 2.93 (3H, s, CH3), 2.36–2.30 (2H, m, CH2), 1.28–1.06 (2H, m, CH2), 0.16 (27H, s, Si[Si(CH3)3]3); δC (CDCl3, 100 MHz), 173.9 (CO), 37.1 (CH3), 35.8 (CH3) 32.5 (*C*H­2CO), 7.0 (*C*H2CH2CO), 1.1 (Si[Si(CH3)3]3); *m/z* (CI+) 348 (5), 332(15), 274 (100), 316 (10), 158 (10); *m/z* (ES+) C14H38NOSi4 (MH+) requires 348.2025, found 348.2025.

***tert*-Butyl (1*R*,8*R*,9*S*)-9-hydroxy-11-azatricyclo[6.2.1.02,7]undeca-2,4,6-triene-11-carboxylate** (**15)**

To (−)-Ipc2BH (1.00 g, 3.5 mmol) at 0 °C was added a solution of alkene **14** [4] (0.570 g, 2.2 mmol) in THF (3 mL) and the solution was stirred for 24 h. MeOH (1 mL) was then added, followed by NaOH (2.0 M aq., 5 mL) and H2O2 (30% w/w aq., 5 mL). The solution was then refluxed for 5 h before being extracted with Et2O (3 × 20 mL), washed (saturated aqueous K2CO3), dried (MgSO4) and the solvent removed under reduced pressure. Column chromatography (50–60% Et2O in petroleum ether) gave alcohol (−)-**15** as a white solid (0.510 g, 84%). [α]25D = −16.1 (CHCl3, *c* = 1.00) *R*f (9:1 Et2O : petroleum ether, silica plates pre-eluted with Et3N) 0.09; max (thin lm) 3500–3050br m, 2924s, 2853s, 1688m, 1461s, 1378s, 1254w, 1154m, 1095w, 952m, 758m, 650w; H (CDCl3, 400 MHz) 7.35–7.01 (4H, m, 4 × aromatic CH), 5.11 (1H, s, NCH), 5.00 (1H, s, NCH), 4.05–3.95 (1H, m, C*H*OH), 1.91–1.87 (2H, m, CH2), 1.40 (9H, s, C(CH3)3); C (CDCl3, 100 MHz) 156.5 (CO), 146.7 (quat. aromatic), 144.4 (quat. aromatic), 127.1 (aromatic CH), 126.5 (aromatic CH), 121.3 (aromatic CH), 119.6 (aromatic CH), 80.5 (*C*(CH3)3), 73.1 (CHOH), 69.0 (NCH), 60.9 (NCH), 39.7 (CH2), 28.2 (C(*C*H3)3). The er was determined to be 97:3 by HPLC analysis: Chiralcel OD column; 0.9 mL min-1; eluent 99% heptane : 1% ethanol; *t*r (major) = 24.30 min, *t*r (minor) = 26.55 min.

***tert*-Butyl (1*R*,8*R*,9*S*)-9-hydroxy-4,5-dimethoxy-11-azatricyclo[6.2.1.02,7]undeca-2,4,6-triene-11-carboxylate (17)**

To (−)-Ipc2BH (1.49 g, 5.2 mmol) at 0 °C was added dropwise a solution of cycloadduct **16** [4] (1.00 g, 3.3 mmol) in THF (5 mL), and the mixture was stirred at 0 °C for 3 d. MeOH (2.3 mL) was added to quench any unreacted borane, followed by NaOH (2.0 M, 7.5 mL) and H2O2 (35% w/w aq., 7.5 mL). The mixture was refluxed for 5 h and then cooled. The mixture was washed (saturated aqueous K2CO3) then extracted with Et2O (3 × 20 mL), dried (MgSO4) and the solvent removed under reduced pressure. Column chromatography (gradient elution, 50–100% Et2O in petroleum ether) gave dimethoxy-substituted alcohol **17** as a yellow solid (0.887 g, 84%). *R*f (1:1 Et2O : petroleum ether) 0.01; [α]25D +16.1 (*c* = 1.0, CHCl3) νmax (Nujol) 3412w br (OH str), 2923s (CH str), 1678w (C=O str), 1083w (C-O str), 1052w (C-O str); δH (400 MHz, CDCl3) 6.91 (1H, s, aromatic CH), 6.83 (1H, s, aromatic CH), 5.06 (1H, d, *J* = 4, NCH), 4.93 (1H, s, NCH), 3.95 (1H, dd, *J* = 7 and 3, C*H*OH), 3.88 (3H, s, OCH3), 3.87 (3H, s, OCH3), 1.90–1.78 (2H, m, CH2), 1.40 (9H, s, C(CH3)3); δC (100 MHz, CDCl3) 156.8 (CO), 148.4 (2 × quat. aromatic), 147.7 (2 × quat. aromatic), 105.9 (aromatic CH), 104.5 (aromatic CH), 80.6 (*C*(CH3)3), 73.4 (CHOH), 61.1 (NCH), 56.2 (NCH), 40.6 (CH2), 28.2 (C(*C*H3)3). The er was determined following oxidation to the corresponding ketone (see below).

***tert*-Butyl (1*R*,8*R*)-4,5-dimethoxy-9-oxo-11-azatricyclo[6.2.1.02,7]undeca-2,4,6-triene-11-carboxylate**

To dimethoxy-substituted alcohol (+)-**17** (200 mg, 0.62 mmol) and *N*-methylmorpholine *N*-oxide (108 mg, 0.92 mmol) in CH2Cl2 (2 mL) were added powdered molecular sieves (4 Å) and TPAP (12 mg, 0.89 mmol). The mixture was stirred at rt for 2.5 h during which time the starting material was consumed (monitored by TLC). The mixture was filtered through celite (eluting with EtOAc) and the solvent removed under reduced pressure. Column chromatography (50% Et2O in petroleum ether) gave the title ketone as a yellow solid (49 mg, 79%). *R*f (1:1 Et2O : petroleum ether) 0.24; [α]25D (CHCl3, *c* = 1.00) +257.4; mp 132–133 °C; νmax (Nujol) 2923m (CH str), 1753s (C=O str), 1710s (C=O str), 1086m (C-O str), 1066m (C-O str); δH (CDCl3, 400 MHz) 6.99 (1H, s, aromatic CH), 6.93 (1H, s, aromatic CH), 5.38 (1H, d, *J* = 4, NCH), 4.94 (1H, s, NCH), 3.89 (3H, s, OCH3), 3.88 (3H, s, OCH3), 2.58 (1H, dd, *J* = 16 and 4, C*H*H), 1.98 (1H, d, *J* = 16, CH*H*), 1.42 (9H, s, C(CH3)3); δC (CDCl3, 100 MHz) 204.7 (CO), 155.1 (CO), 149.4 (*C*OCH3), 148.6 (*C*OCH3), 139.0 (quat. aromatic), 129.2 (quat. aromatic), 106.8 (aromatic CH), 105.0 (aromatic CH), 81.4 (*C*(CH3)3), 69.3 (NCH), 61.1 (NCH), 56.2 (OCH3), 56.2 (OCH3), 39.5 (CH2), 28.1 (C(*C*H3)3); *m/z* (CI+) 320 (MH+, 2%), 281 (8), 264 (60), 220 (100), 203 (47), 190 (100), 177 (6), 131 (4), 87 (3), 70 (4); *m/z* (CI+) C17H22NO5 (MH+) requires 320.1492, found 320.1487. The er was determined to be >99:1 by HPLC analysis: Chiralcel OD column; 0.2 mL min-1; eluent 99% heptane : 1% ethanol; *t*r (major) = 112 min, *t*r (minor – not observed in enantioenriched product) = 139 min.

***tert*-Butyl (1*R*,8*R*,9*S*)-9-hydroxy-1,8-dimethyl-11-azatricyclo[6.2.1.02,7]undeca-2,4,6-triene-11-carboxylate (19)**

To (−)-Ipc2BH (10.2 g, 35 mmol) at 0 °C was added dropwise a solution of cycloadduct **18** [6,7] (6.06 g, 22 mmol) in THF (40 mL) and the mixture stirred for 3 d at 0 °C. Methanol (10 mL) was then added, followed by aqueous NaOH (2 M, 50 mL) and H2O2 (35% w/w, 50 mL). The mixture was refluxed for 5 h and then cooled to rt. The mixture was washed (saturated aqueous K2CO3), extracted with Et2O (3 × 50 mL), dried (MgSO4) and the solvent removed under reduced pressure. Column chromatography (gradient elution, 10–60% Et2O in petroleum ether) gave alcohol **19** as a colourless solid (5.00 g, 67%). *R*f (1:9 Et2O : petroleum ether) 0.16; mp (Et2O) 64–65 °C; [α]25D +30.1 (CHCl3, *c* = 1.00); νmax (Nujol) 3427br m (OH str), 2923s (CH str), 1658s (C=O str), 1072m (C-O str); δH (CDCl3, 400 MHz), 7.08–6.83 (4H, m, 4 × aromatic CH), 3.70 (1H, dd, *J* 7 and 2, C*H*OH), 2.04–1.98 (6H and 1H, s and obscured m, 2 × CH3 and C*H*H), 1.72 (1H, m, C*H*H), 1.42 (9H, s, C(CH3­)3); δC (CDCl3, 100 MHz) 156.8 (CO), 149.0 (quat. aromatic), 145.1 (quat. aromatic), 127.1 (aromatic CH), 126.6 (aromatic CH), 119.4 (aromatic CH), 117.5 (aromatic CH), 80.4 (*C*(CH3)3), 76.8 (CHOH), 73.3 (*C*CH3), 65.9 (*C*CH3), 47.4 (CH2), 28.4 (C(*C*H3)3), 17.6 (C*C*H3), 13.6 (C*C*H3). The er was determined to be >99:1 by GC analysis: Chirasil Dex-CD column; 1.0 mL min-1;*t*r (minor – not observed in the enantioenriched product) = 540 min, *t*r (major) = 544 min.

***tert*-Butyl (1*R*,8*R*,9*S*)-9-[(methylsulfanyl)thiocarbonyloxy]-4,5-dimethoxy-11-azatricyclo[6.2.1.02,7]undeca-2,4,6-triene-11-carboxylate (20)**

To KH (30% suspension in mineral oil, 3.53 g, 26 mmol) in THF (60 mL) at 0 °C was added dropwise a solution of alcohol (+)-**17** (2.61 g, 6.7 mmol) in THF (20 mL). The mixture was stirred at rt for 20 min, then cooled to 0 °C. CS2 (1.6 mL, 27 mmol) was added, and the mixture stirred for 10 min. MeI (1.6 mL, 27 mmol) was added and the mixture allowed to warm to rt over 30 min. Excess KH was quenched by the cautious dropwise addition of water until effervescence ceased and the solution became translucent (*ca*. 5 mL). The mixture was extracted with Et2O (3 × 50 mL), dried (MgSO4) and the solvent removed under reduced pressure. Column chromatography (20% Et2O in petroleum ether) gave dimethoxy-substituted xanthate **20** as a yellow foam (3.21 g, 96%, lit. (racemate) [4] 77%). *R*f (1:1 Et2O : petroleum ether) 0.22; [α]25D +33.5 (CHCl3, *c* = 1.00); νmax (Nujol) 2923s (CH str), 1700s (C=O str), 1203s (C=S str); δH (CDCl3, 400 MHz) 6.95 (1H, s, aromatic CH), 6.84 (1H, s, aromatic CH), 5.37–5.27 (2H, m, 2 × NCH), 5.22–5.01 (1H, br s, C*H*OCS2CH3), 3.86 (3H, s, OCH3), 3.84 (3H, s, OCH3), 2.56 (3H, s, SCH3), 2.18–2.07 (1H, m, C*H*H), 1.91 (1H, dd, *J* = 13 and 7, CH*H*), 1.41 (9H, s, C(CH3)3); δC (CDCl3, 100 MHz), 215.6 (CS), 154.5 (CO), 148.5 (quat. aromatic), 147.8 (quat. aromatic), 138.5 (quat. aromatic), 132.1 (quat. aromatic), 105.9 (aromatic CH), 103.3 (aromatic CH), 85.0 (NCH), 80.4 (*C*(CH3)3), 65.5 (NCH), 56.2 (2 × OCH3), 36.2 (CH2), 28.2 (C(*C*H3)­3), 19.2 (SCH3).

***tert*-Butyl (1*R*,8*S*)-4,5-dimethoxy-9-azatricyclo[6.2.1.02,7]undeca-2,4,6-triene-9-carboxylate**

**(21)** [4]

Dimethoxy-substituted xanthate (+)-**20** (250 mg, 0.61 mmol) was dissolved in toluene (15 mL) and heated to reflux. (TMS)3SiH (0.28 mL, 0.91 mmol) and AIBN (51 mg, 0.31 mmol) in toluene (4 mL) were added *via* syringe pump over 100 min. The reaction mixture was allowed to reflux for a further 30 min before being cooled to rt and the solvent removed under reduced pressure. Column chromatography (40% Et2O in petroleum ether) gave rearranged-reduced dimethoxy-substituted azacycle **21** as a yellow oil (130 mg, 70%, lit. (racemate) [4] 78%). *R*f (2:3 Et2O : petroleum ether) 0.24; H (CDCl3, 200 MHz) 7.07–6.83 (2H, m, 2 × aromatic CH), 5.10 and 4.90 (0.35H and 0.65H, s and s, NCH), 3.82 (6H, s, 2 × OCH3), 3.62–3.42 (2H, m, C*H*H and CH), 2.81–2.62 (1H, m, CH*H*), 2.40–1.80 (2H, m, CH2), 1.51 and 1.48 (3.2H and 5.8H, s and s, rotamers, C(CH3)3). On standing (12 h), **21** isomerised to the corresponding indene **22**. [α]25D +112.6 (CHCl3, *c* = 1.00); δH (CDCl3, 400 MHz) 7.05 (1H, s, aromatic CH), 6.94 (1H, s, aromatic CH), 6.79 (1H, d, *J* = 5, vinylic CH), 6.41 (1H, d, *J* = 5, vinylic CH), 4.43 (1H, s, NH), 3.92 (6H, s, 2 × OCH3), 3.74–3.60 (2H, m, CH and C*H*H), 3.34–3.26 (1H, m, CH*H*), 1.42 (C(CH3)3); δC (CDCl3, 100 MHz) [CO not observed] 148.5 (quat. aromatic), 147.4 (quat. aromatic), 137.3 (quat. aromatic), 137.0 (quat. aromatic), 135.4 (vinylic CH), 132.3 (vinylic CH), 107.3 (aromatic CH), 104.8 (aromatic CH), 79.1 (*C*(CH3)3), 56.2 (OCH3), 56.1 (OCH3), 50.6 (CH), 48.9 (CH2), 28.4 (C(*C*H3)3).

***tert*-Butyl (1*R*,8*R*,9*S*)-9-[(methylsulfanyl)thiocarbonyloxy]-1,8-dimethyl-11-azatricyclo[6.2.1.02,7]undeca-2,4,6-triene-11-carboxylate** (**23)**

To KH (30% suspension in mineral oil, 7.93 g, 59 mmol) in THF (150 mL) at 0 °C was added dropwise a solution of alcohol (+)-**19** (4.31 g, 15 mmol) in THF (40 mL). The mixture was stirred at rt for 20 min, then cooled to 0 °C. CS2 (3.6 mL) was added, and the mixture stirred for 10 min. MeI (3.7 mL) was added and the mixture allowed to warm to rt over 30 min. Excess KH was quenched by the cautious dropwise addition of water until effervescence ceased and the solution became translucent (*ca*. 5 mL). The mixture was extracted with Et2O (3 × 50 mL), dried (MgSO4) and the solvent removed under reduced pressure. Column chromatography (20% Et2O in petroleum ether) gave xanthate **23** as a yellow oil that crystallised on standing to a glassy yellow solid (5.42 g, 96%). *R*f (1:4 Et2O : petroleum ether) 0.47; mp (Et2O) 82 °C; [α]25D +75.0 (CHCl3, *c* = 1.00); νmax (Nujol) 2923s (CH str), 1695m (C=O str), 1208m (C-S str), 1067m (C-O str); δH (CDCl3, 400 MHz) 7.29–7.13 (4H, m, 4 × aromatic CH), 5.38 (1H, dd, *J* = 7 and 2, CHO), 2.60 (3H, s, SCH3), 2.16 (1H, dd, *J* = 13 and 7, C*H*H), 2.06 (3H, s, CH3), 2.03 (3H, s, CH3), 1.97 (1H, dd, *J* = 13 and 2, CH*H*), 1.48 (9H, s, C(CH3)3); δC­ (CDCl3, 100 MHz), 215.5 (CS), 155.0 (CO), 149.0 (quat. aromatic), 143.8 (quat. aromatic), 127.7 (aromatic CH), 126.9 (aromatic CH), 119.6 (aromatic CH), 117.7 (aromatic CH), 87.8 (CHO), 80.3 (*C*(CH3)3), 72.0 (*C*CH3), 67.9 (*C*CH3), 44.2 (CH2), 28.5 (C(*C*H3)3), 19.2 (SCH3), 16.9 (C*C*H3), 14.2 (C*C*H3).

***tert*-Butyl {(*R*)-1-[(*S*)-3-methyl-1*H*-inden-1-yl]ethyl}carbamate (25)**

To xanthate (+)-**23** (250 mg, 0.65 mmol) in refluxing toluene (20 mL) was added AIBN (54 mg, 0.33 mmol) and (TMS)3SiH (246 mg, 0.99 mmol) in toluene (5 mL) over 100 min *via* syringe pump. The mixture was stirred at reflux for a further 30 min, then cooled to rt and the solvent removed under reduced pressure. Column chromatography (10% Et2O in petroleum ether) gave indene **25** [6]as a colourless oil that crystallised on standing as a white solid (108 mg, 60%). *R*f (4:1 Et2O : petroleum ether) 0.40; [α]25D +42.2 (CHCl3, *c* = 1.00); νmax (KBr) 3370s, 2981s, 2938m, 1638s, 1523s, 1455m, 1381m, 1366m, 1339m, 1250m, 1170s, 1081m, 1050m; δH­­ (400 MHz, CDCl3) 7.49–7.43 (1H, m, aromatic CH), 7.36–7.26 (2H, m, 2 × aromatic CH), 7.23–7.16 (1H, m, aromatic CH), 6.10 (1H, m, vinylic CH), 4.57–4.47 (1H, m, NH), 4.29 (1H, s, C*H*CH3), 3.71 (1H, s, CH), 2.16–2.14 (3H, m, C=CCH3), 1.47 (9H, s, C(CH3)­3), 0.86 (3H, d, *J* = 7, CH­3); δC (100 MHz, CDCl3) 155.3 (CO), 146.2 (quat. aromatic), 144.8 (quat. aromatic), 141.1 (quat. vinylic), 130.0 (vinylic CH), 126.8 (aromatic CH), 124.9 (aromatic CH), 123.2 (aromatic CH), 119.0 (aromatic CH), 79.3 (*C*(CH3)3), 53.9 (*C*HC=C), 47.7 (NCH), 28.5 (C(*C*H3)3), 16.7 (CH3), 13.1 (CH3); *m/z* (ES+) 274 ([MH]+, 9%), 259 (27), 229 (4), 218 (100), 172 (9), 157 (67); *m/z* (ES+) C17H24NO2 (MH+) requires 274.1807, found 274.1802.

*t****ert*-Butyl {(*S*)-3-cyano-1-[(*S*)-5,6-dimethoxy-1*H*-inden-1-yl]propyl}carbamate (27)**

To dimethoxy-substituted xanthate (+)-**20** (250 mg, 0.61 mmol) in refluxing toluene (20 mL) was added dropwise over 90 min (*via* syringe pump) a solution of AIBN (50 mg, 0.30 mmol), (TMS)3SiH (0.28 mL, 0.91 mmol) and acrylonitrile (0.06 mL, 0.91 mmol). The reaction was stirred for a further 1 h at reflux and then cooled to rt. The solvent was removed under reduced pressure [diagnostic features for **26** were observed: δH 5.04 (s, NCH) and 3.63 (s, CH)]. Initial column chromatography (gradient elution, 10–30% Et2O in petroleum ether) gave impure amino indene **27** (28 mg, ~17%) as a colourless oil that further decomposed on standing and during subsequent column chromatography. *R*f (1:4 Et2­O : petroleum ether) 0.08. Indene **27** decomposed before full characterisation could be obtained, but was assigned on the basis of characteristic chemical shifts: δH (CDCl3, 400 MHz) 6.88–6.84 (1H, m, C*H*=CH), 6.30–6.26 (1H, m, CH=C*H*), 4.69 (1H, s, NH).

***tert*-Butyl {(*S*)-4-cyano-2-[(*S*)-3-methyl-1*H*-inden-1-yl]butan-2-yl}carbamate (29)**

To xanthate (+)-**23** (250 mg, 0.66 mmol) in refluxing toluene (20 mL) was added dropwise over 90 min (*via* syringe pump) a solution of AIBN (54 mg, 0.33 mmol), (TMS)3SiH (0.31 mL, 0.99 mmol) and acrylonitrile (0.06 mL, 0.99 mmol). The reaction was stirred for a further 1 h at reflux and then cooled to rt. The solvent was removed under reduced pressure. NMR and GC-LRMS analysis
of the crude product mixture indicated diagnostic features for **28** (R = CN, δH 3.19 (s, CH), 2.25 (d, *J* = 9.6, C*H*H), 1.74 (d, *J* = 9.6, CH*H*)) and the yield was estimated as ≤59%. Initial column chromatography (gradient elution, 10–40% Et2O in petroleum ether) gave impure amino indene **29** (24 mg, 11%) as a colourless oil that decomposed on standing and during subsequent column chromatography. *R*f (1:4 Et2O : petroleum ether) 0.11. Indene **29** decomposed before full characterisation could be obtained, but was assigned on the basis of characteristic 1H NMR chemical shifts: δH (CDCl3, 400 MHz) 6.08 (1H, s, C*H*=CCH3), 4.69 (1H, s, NH).

**Methyl (*S*)-4-(*tert*-butoxycarbonylamino)-4-[(*S*)-3-methyl-1*H*-inden-1-yl]pentanoate (30)**

To xanthate (+)-**23** (250 mg, 0.66 mmol) in refluxing toluene (20 mL) was added dropwise over 90 min (*via* syringe pump) a solution of AIBN (54 mg, 0.33 mmol), (TMS)3SiH (0.31 mL, 0.99 mmol) and methyl acrylate (0.09 mL, 0.99 mmol). The reaction was stirred for a further 1 h at reflux and then cooled to rt. The solvent was removed under reduced pressure. Careful column chromatography (gradient elution, 5–40% Et2O in petroleum ether) gave amino indene **30** as a colourless oil (57 mg, 28%). *R*f (1:4 Et2O : petroleum ether) 0.11; [α]25D +15.4 (*c* = 1.00, CHCl3); νmax (thin film) 3379w, 2975m, 2361w, 1737s, 1694s, 1499m, 1478m, 1456m, 1439s, 1293w, 1251m, 1167s, 1068m, 1051m, 848w, 803w, 760w, 730w, 698w; δH (CDCl3, 400 MHz) 7.50–7.43 (1H, m, aromatic CH), 7.37–7.24 (2H, m, 2 × aromatic CH), 7.21–7.11 (1H, m, aromatic CH), 6.09 (1H, s, vinylic CH), 4.92 (1H, s, NH), 4.37 (1H, s, CHC=C), 3.70 and 3.64 (2.5H and 0.5H, s and s, rotamers, OCH3), 2.37–2.21 (2H, m, C*H*2CO2CH3) 2.05–1.93 (1H, m, C*H*H), 1.83–1.68 (1H, m, CH*H*), 1.54 (3H, s, CH3), 1.50 (9H, s, C(CH3)3), 1.36 (3H, s, CH3); δC (CDCl3, 100 MHz) 174.3 and 172.8 (CO), 154.6 (CO) 146.7 (quat. aromatic), 144.6 and 140.5 (rotamers, quat. aromatic), 130.9 (C=*C*H), 128.4 (C=*C*CH3), 124.8 (aromatic CH) 124.1 (aromatic CH), 119.1 (2 × aromatic CH), 79.2 (*C*(CH3)3), 58.8 (N*C*CH3), 51.9 and 51.7 (OCH3), 39.4 (*C*C=C), 35.7 (CH2), 28.7 (CH2), 28.5 (C(*C*H3)3), 26.5 (CH3), 23.4 (CH3).

***tert*-Butyl {(*S*)-2-[(*S*)-3-methyl-1*H*-inden-1-yl]-4-(phenylsulfonyl)butan-2-yl}carbamate (31)**

To xanthate (+)-**23** (250 mg, 0.66 mmol) in refluxing toluene (20 mL) was added dropwise over 90 min *via* syringe pump a solution of AIBN (54 mg, 0.33 mmol), (TMS)3SiH (0.31 mL, 0.99 mmol) and phenyl vinyl sulfone (167 mg, 0.99 mmol). The reaction was stirred for a further 1 h at reflux and then cooled to rt. The solvent was removed under reduced pressure. Careful column chromatography (gradient elution, 5–60% Et2O in petroleum ether) gave amino indene **31** as a pale yellow oil (80 mg, 28%). *R*f (2:3 Et2O : petroleum ether) 0.20; [α]25D +6.1 (CHCl3, *c* = 1.00); νmax (thin film) 3356w, 2924s, 2360w, 1710s, 1514m, 1459s, 13m, 1304m, 1247m, 1149s, 1087m, 1061m, 731m, 689w; δH (CDCl3, 400 MHz) 7.89–7.81 (2H, m, 2 × sulfone aromatic CH), 7.69–7.63 (1H, m, sulfone aromatic CH), 7.60–7.52 (2H, m, 2 × sulfone aromatic CH), 7.36–7.23 (3H, m, 3 × aromatic CH), 7.19–7.13 (1H, m, aromatic CH), 6.02 (1H, s, vinylic CH), 4.67 (1H, s, NH), 4.23 (1H, s, CH), 3.16–3.00 (2H, m, CH2SO2), 2.34–2.18 (1H, m, C*H*H) 2.12–2.09 (3H, m, CH3); 2.05–1.90 (1H, m, CH*H*) 1.45 (9H, s, C(CH3)3), 1.05 (3H, s, CH3); δC (CDCl3, 100 MHz) 154.5 (CO), 146.8 (quat. aromatic), 143.8 (quat. aromatic), 141.1 (quat. aromatic), 138.8 (quat. vinylic), 133.8 (sulfone aromatic CH), 130.3 (vinylic CH), 129.3 (2 × sulfone aromatic CH), 128.0 (2 × sulfone aromatic CH), 126.9 (aromatic CH) 125.0 (aromatic CH), 124.0 (aromatic CH), 119.3 (aromatic CH), 79.6 (*C*(CH3)3, 56.4 (CH), 55.7 (*C*NCH3), 52.0 (SO2CH2), 30.3 (SO2CH2*C*H2), 28.4 (C(*C*H3)3), 22.3 (CH3), 13.0 (CH3); *m/z* (CI+) 459 (10), 442 (MH+, 15), 403 (35), 386 (12), 368 (7), 342 (12), 300 (13), 263 (10), 245 (25), 200 (52), 131 (50), 94 (42), 70 (100); *m*/*z* (CI+) C25H32O4NS (MH+) requires 442.2047, found 442.2050.

***tert*-Butyl (1*S*,8*R*)-9-azatricyclo[6.2.1.02,7]undeca-2,4,6-triene-9-carboxylate (8)**

To xanthate (+)-**5** [3] (250 mg, 0.71 mmol) in refluxing toluene (21 mL) was added AIBN (60 mg, 0.36 mmol) and (TMS)3SiH (266 mg, 1.1 mmol) in toluene (2 mL) over 100 min *via* syringe pump. The mixture was stirred at reflux for a further 30 min, then cooled to rt and the solvent removed under reduced pressure. Column chromatography (10% Et2O in petroleum ether) gave rearranged–reduced azacycle **8** as a colourless oil that crystallised on standing to a white solid (166 mg, 97%). *R*f (1:4 Et2O : petroleum ether) 0.20; [α]25D +97.5 (CHCl3, *c* = 1.00); mp 65–67 °C (lit. racemate [4] 65–66 °C); νmax (thin film) 2978s, 1695s, 1461w, 1391m, 1310w, 1250w, 1181w, 1146m, 1014w, 901w, 883w, 867w, 834w; δH (CDCl3, 400 MHz) 7.31–7.26 (2H, m, 2 × aromatic CH), 7.19–7.10 (2H, m, 2 × aromatic CH), 5.12 and 4.96 (0.5H and 0.5H, s and s, rotamers, NCH), 3.62 (1H, s, CH), 3.56–3.50 (1H, m, NC*H*H), 2.89–2.70 (1H, m, NCH*H*), 2.05–1.93 (1H, m, C*H*H), 1.91–1.86 (1H, m, CH*H*), 1.44 (9H, s, C(CH3)3); C (CDCl3, 100 MHz), 154.9 (CO), 146.2 (quat. aromatic), 144.9 (quat. aromatic), 126.8 (aromatic CH), 126.1 (aromatic CH), 122.2 (aromatic CH), 121.5 (aromatic CH), 79.2 (*C*(CH3)3), 62.4 and 67.2 (rotamers, NCH), 48.4 (NCH2), 48.3 (CH2), 44.5 (CH), 28.5 (C(*C*H3)3).

**1-*tert*-Butyl 2,4-dimethyl (2*R*,4*R*)-pyrrolidine-1,2,4-tricarboxylate (35)**

Azacycle (+)-**8** (250 mg, 1.0 mmol) in EtOAc (16 mL) was added dropwise to a vigorously stirred mixture of NaIO4 (3.78 g, 18 mmol) and RuCl3·3H2O (25.5 mg, 0.10 mmol) in water (32 mL) and MeCN (16 mL) in an open vessel *(caution: large volumes of CO2 evolved)*. The reaction mixture was stirred vigorously for 4 h, by which time a white precipitate had formed. Et2O was added dropwise until the solution turned black (*ca.* 1 mL). The mixture was filtered through celite, extracted with EtOAc (3 × 20 mL), dried (MgSO4), filtered through celite and the solvent removed under reduced pressure to afford 230 mg of crude diacid. The residue was dissolved in Et2O (10 mL). To half of the diacid solution in Et2O was added dropwise an ethereal solution of diazomethane [freshly generated with a Sigma-Aldrich Mini-Diazald Kit, using Diazald (2.5 g, 11.5 mmol)] and stirred for 1.5 h. Removal of the solvent under reduced pressure followed by column chromatography gave diester **35** as a yellow oil (71 mg, 49% from **8**). *R*f (1:1 Et2O : petroleum ether) 0.24; [α]25D +38.3 (CHCl3, *c* = 1.00); νmax (thin film) 2977m, 1742s, 1703s, 1437m, 1402s, 1163s, 1119m, 1061w, 1028w, 991w, 929w, 898w, 866w, 773w; δH (CDCl3, 400 MHz) 4.37–4.22 (1H, m, NCH(CO2Me)), 3.88–3.73 (1H, m, NC*H*H), 3.72–3.62 (7H, m, 2 × OCH3 and NCH*H*), 3.12–2.98 (1H, m, CH), 2.52–2.43 (1H, m, C*H*H), 2.37–2.25 (1H, m, CH*H*), 1.48–1.36 (9H, m, C(CH3)3); δC (CDCl3, 100 MHz) 172.8 and 172.5 (rotamers, *C*O2CH3), 172.2 (*C*O2CH3), 153.9 and 153.3 (rotamers, NCO), 80.3 (*C*(CH3)3), 58.7 and 58.3 (rotamers, NCH), 52.2 (OCH3), 52.0 (OCH3), 48.6 and 48.4 (rotamers, NCH2), 42.3 and 41.6 (rotamers, CH2*C*HCH2), 33.0 and 32.1 (rotamers, CH2), 28.3 and 28.2 (rotamers, C(*C*H3)3); *m/z* (CI+) 288 (MH+, 10%), 249 (20), 188 (100), 128 (15), 52 (15); *m*/*z* (ES+) C13H22NO6 (MH+) requires 288.1442, found 288.1441. The remaining crude diacid was evaporated to dryness under reduced pressure and then dissolved in PhMe (7 mL) and MeOH (2 mL). TMSCHN2 (0.48 mL, 2.0 M in Et2O, 1.0 mmol) was added dropwise and the reaction stirred for 45 min. Removal of the solvent under reduced pressure followed by column chromatography (40–60% Et2O in petroleum ether) gave diester **35** as a yellow oil (53 mg, 37% from **8**). Spectral data as above.

**1-*tert*-Butyl 2,4-dimethyl (2*R*,4*R*,5*S*)-5-(3-methoxy-3-oxopropyl)pyrrolidine-1,2,4-tricarboxylate (36)**

To NaIO4 (1.14 g, 0.75 mmol) in MeCN/H2O (1:2, 15 mL) was added RuCl3·3H2O (7.5 mg, 0.029 mmol). Methoxyoxopropyl-substituted 2-azabenzonorbornene (+)-**10** (100 mg, 0.30 mmol) was then added in EtOAc (5 mL) and the reaction stirred in an open flask for 5 h. The mixture was filtered through celite, extracted (EtOAc, 3 × 25 mL), dried (MgSO4) and the solvent removed under reduced pressure. The crude diacid was then dissolved in PhMe/MeOH (3:2, 5 mL) and TMSCHN2 was added dropwise (0.30 mL, 2.0 M in Et2O, 0.6 mmol). The mixture was stirred for 40 min and the solvent removed under reduced pressure. Column chromatography (gradient elution, 40–60% Et2O in petroleum ether) gave pyrrolidine **36** as a yellow oil (26 mg, 28%). *R*f (1:1 Et2O : petroleum ether) 0.09; [α]25D +5.8 (CHCl3, *c* = 1.00); νmax (thin film) 3584w, 3453w, 2955s, 2361w, 1736s, 1699s, 1478w, 1437w, 1390m, 1367w, 1324w, 1282w, 1256m, 1170w, 1122m, 1063w, 1038w; δH (CDCl3, 400 MHz); 4.86 (1H, dd, *J* = 10 and 3, NC*H*CO2Me), 4.29–4.17 (2H, m, NCH and C*H*CO­2Me); 3.63–3.60 (6H, m, 2 × OCH3), 3.08–2.96 (3H, s, OCH3), 2.42–2.30 (2H, m, CH2), 2.12–2.00 (2H, m, CH2C*H2*CO2Me), 1.92–1.78 (2H, m, *CH2*CH2CO2Me), 1.38 and 1.36 (5H and 4H, s and s, rotamers, C(CH3)3); δC (CDCl3, 100 MHz) 173.2 and 173.1 (rotamers, CH2CH2*C*O2Me), 172.7 (CO2Me) 172.6 (CO2Me), 161.0 (CO2*t*Bu), 80.5 and 79.9 (rotamers, *C*(CH3)3), 62.9 (NCH), 60.8 and 60.3 (rotamers, NCH2), 59.5 (*C*HCO2Me), 55.1 (CH3), 53.2 and 52.3 (rotamers, CH­3), 51.0 and 51.6 (rotamers, CH­3), 32.0 (CH2), 30.5 and 30.3 (rotamers, CH2), 29.3 and 29.0 (rotamers, CH2), 28.4 and 28.3 (rotamers, C(*C*H3)3); *m/z* (CI+) 374 (30), 363 (32), 346 (30), 335 (100), 318 (55), 274 (72), 242 (43), 214 (63); *m/z* (ES+) C17H28NO8 (MH+) requires 374.1809, found 374.1808.

***tert*-Butyl (1*R*,4*S*)-2-azabicyclo[2.2.1]hept-5-ene-2-carboxylate (38)** [8,9]

To a stirred solution of (1*R*)-(−)-2-azabicyclo[2.2.1]hept-5-en-3-one (Aldrich, 200 mg, 1.8 mmol) in THF/Et2O (5:1, 12 mL) was added dropwise LiAlH4 (9 mL, 1.0 M in Et2O, 9.0 mmol). The mixture was heated at reflux for 7 h, during which time a white precipitate formed. After cooling to 0 °C in an ice bath, water (3 mL) was added with vigorous stirring. The mixture was filtered through celite. Di-*tert*-butyl dicarbonate (446 mg, 2.0 mmol) was added and the reaction was stirred for 14 h. PhMe was added (20 mL) and the mixture was dried (MgSO4) and the solvent was removed under reduced pressure. Column chromatography (10% EtOAc in petroleum ether) gave azacycle **38** [10] as a colourless oil (283 mg, 79%). [α]25D +9.6 (CHCl3, *c* = 0.3); δH (CDCl­3, 200 MHz) 6.45–6.22 (2H, m, vinylic CH), 4.71 and 4.58 (0.45H and 0.55H, s and s, NCH), 3.31 (1H, dd, *J* = 9.0 and 2.9, NC*H*H), 3.21–3.12 (1H, m, CH), 2.69–2.51 (1H, m, NCH*H*­), 1.62–1.50 (1H, m, CH2), 1.46 (9H, s, C(CH3)3).

**1-*tert*-Butyl 2,4-dimethyl (2*R*,4*R*)-pyrrolidine-1,2,4-tricarboxylate (35)**

A solution of azacycle **38** (100 mg, 0.35 mmol) in EtOAc (4 mL) was added to a pre-cooled stirred solution of RuCl3·H2O (10 mg, 0.05 mmol) and NaIO4 (1.20 g) in water (11 mL), at 0 °C, and the mixture stirred at 0 °C for 8 h. The resulting mixture was filtered through celite and extracted with EtOAc (3 × 20 mL). The combined organic extracts were dried (MgSO4) and concentrated under reduced pressure. The residue was dissolved in PhMe (10 mL) and MeOH (3 mL). TMSCHN2 was added (0.35 mL, 2.0 M in Et2O, 0.7 mmol) and the mixture stirred for 45 min. Concentration under reduced pressure, followed by column chromatography (40–60% Et2O in petroleum ether) gave pyrrolidine **1** as a yellow oil (44 mg, 37%). [α]25D +21.6 (*c* = 1.0, CHCl3); other characterisation as above.

***tert*-Butyl [(3-hydroxy-2,3-dihydro-1*H*-inden-1-yl)methyl]carbamate (mixture of diastereomers)** (**33)**

To azacycle (+)-**8** (100 mg, 0.41 mmol) in THF (2 mL) was added 2 M aq. HCl (2 mL) and the solution stirred for 6 h (TLC analysis indicated complete consumption of starting material). The mixture was extracted with EtOAc (2 × 10 mL), dried (MgSO4) and the solvent removed under reduced pressure. Column chromatography (60% Et2O in petroleum ether) gave alcohol **39** as a colourless oil (60 mg, 56%), shown to be an inseparable mixture of diastereomers (approximately 3:2 ratio). *R*f (1:1 Et2O : petroleum ether) 0.04; νmax (thin film) 3350br s, 2977s, 932s, 2247w, 1693s, 1517s, 1478s, 1458s, 1393s, 1367s, 1252s, 1110s, 1082m, 912m, 862w, 757s, 734s; δH (CDCl3, 400 MHz) 7.43–7.38 (1H, m, aromatic CH), 7.32–7.22 (3H, m, 3 × aromatic CH), 5.30–5.11 (1H, m, C*H*OH), 4.95 (0.4H, m, NH), 4.64 (0.6H, m, NH’), 3.65–3.12 (3H, m, NCH2 and CH), 2.85 (0.4H, s, OH), 2.64–2.42 (1H, m, C*H*H and OH’), 2.25–2.05 (1H, m, CH*H* and C*H*H’), 1.77–1.67 (0.6H, m, CH*H*’), 1.43 (6H, s, C(CH3)3), 1.42 (3H, s, C(CH3)3’); δC (CDCl3, 100 MHz) 156.4 (CO), 156.1 (CO’), 145.5 (quat. aromatic), 145.4 (quat. aromatic’), 143.8 (quat. aromatic), 143.5 (quat. aromatic’), 128.6 (aromatic CH), 128.5 (aromatic CH’), 127.6 (aromatic CH), 127.5 (aromatic CH’), 124.7 (aromatic CH), 124.5 (aromatic CH’), 124.3 (aromatic CH), 124.1 (aromatic CH’), 79.4 (*C*(CH3)3), 74.9 (CHOH), 74.7 (CHOH’), 44.5 (NCH2), 42.9 (CH), 42.6 (CH’). 40.1 (CH2), 39.6 (CH2’), 28.4 (C(*C*H3)3); *m/z* (CI+) 281.3 (22%), 264.3 (12), 246.2 (18), 225.1 (32), 207.1 (100), 190.2 (25), 146.1 (50), 116.2 (8); *m/z* (ES+) C15H21O3NNa (MNa+) requires 286.1414, found 286.1414.

***tert*-Butyl [(*S*)-(3-oxo-2,3-dihydro-1*H*-inden-1-yl)methyl]carbamate (34)**

To alcohol **39** (55 mg, 0.21 mmol) in CH2Cl2 (1 mL) was added *N*-methylmorpholine *N*-oxide (37 mg, 0.32 mmol) and powdered molecular sieves (4 Å, 100 mg). TPAP (4 mg, 0.01 mmol) was then added and the reaction stirred for 3 h. The mixture was filtered through celite, eluting with CH2Cl2 (50 mL) then EtOAc (50 mL). The combined fractions were dried (MgSO4) and the solvent removed under reduced pressure. Column chromatography (60% Et2O in petroleum ether) gave ketone **34** as a white solid (44 mg, 80%). *R*f (1:1 Et2O : petroleum ether) 0.15; [α]25D +6.0 (CHCl3, *c* = 0.5); mp 74–75 °C; νmax (Nujol) 2924s (CH str), 2854s (CH str), 1707m (C=O str), 1686m (C=O str); δH (CDCl3, 400 MHz) 7.75 (1H, d, *J* = 8, aromatic CH), 7.64–7.59 (1H, m, aromatic CH), 7.57–7.53 (1H, m, aromatic CH), 7.44–7.38 (1H, m, aromatic CH), 4.67 (1H, s, NH), 3.68–3.57 (2H, m, CH and NC*H*H), 3.37–3.27 (1H, m, NCH*H*), 2.83 (1H, dd, *J* = 19 and 8, C*H*HCO), 2.47 (1H, dd, *J* = 19 and 3, CH*H*CO), 1.42 (9H, s, C(CH3)3); δC (CDCl3, 100 MHz) 205.4 (CO), 156.0 (NCO2), 155.3 (quat. aromatic), 137.4 (quat. aromatic), 134.8 (aromatic CH), 128.1 (aromatic CH), 125.8 (aromatic CH), 123.9 (aromatic CH), 123.8 (aromatic CH), 79.7 (*C*(CH3)3), 44.8 (NCH2), 40.8 (*C*H2CO), 38.7 (CH), 28.3 (C(*C*H3)3); *m/z* (CI+) 279.3 (35%), 223.2 (100), 205.2 (47), 188.1 (8), 179.1 (12), 162.1 (88), 132.1 (11); *m/z* (ES+) C15H19O3NNa (MNa+) requires 284.1257, found 284.1256.

***tert*-Butyl {[(*S*)-2,3,4,7-tetrahydro-1*H*-inden-1-yl]methyl}carbamate** (**39)**

To a mixture of freshly-condensed liquid ammonia (25 mL), THF (1 mL) and EtOH (1 mL) at −78 °C was added azacycle (+)-**8** (300 mg, 1.2 mmol) in THF (1 mL). Sodium pieces (~10 mg each, *ca*. 80 mg in total) were slowly added until a blue colour persisted. Further additions of sodium (*ca*. 60 mg) were made after 1 h (when the blue colour had dissipated) until the blue colour was found to persist again. After 6 h the reaction decolourised again, and TLC analysis indicated complete consumption of starting material. The reaction was quenched by the addition of ammonium chloride (1.00 g, 19 mmol). The mixture was allowed to warm to rt over 16 h, and the organic residue was extracted with Et2O (3 × 30 mL), dried (MgSO4) and the solvent removed under reduced pressure. Column chromatography (gradient elution, 10–30% Et2O in petroleum ether) gave diene **39** as a colourless oil that crystallised on standing to a white solid (161 mg, 53%). *R*f (1:1 Et2O : petroleum ether) 0.64; mp 47–48 °C; [α]25D +15.0 (CHCl3, *c* = 1.00); νmax (Nujol) 3390s (NH str), 2925s (CH str), 2852s (CH str), 1688s (C=O str); δH (CDCl3, 400 MHz) 5.75 (2H, s, 2 × vinylic CH), 4.45 (1H, br s, NH), 3.42–3.29 (1H, m, NHC*H*H), 3.04–2.96 (1H, m, NHCH*H*), 2.78–2.49 (5H, m, CH2C=CCH2 and CH), 2.35–2.14 (2H, m, CH2), 2.09–1.97 (1H, m, CHC*H*H), 1.63–1.52 (1H, m, CHCH*H*), 1.44 (9H, s, C(CH3)3); δC (CDCl3, 100 MHz) 156.2 (CO), 134.7 (quat. vinylic), 131.6 (quat. vinylic), 124.4 (H*C*=*C*H), 79.1 (*C*(CH3)3), 47.6 (CH), 42.9 (NCH2), 34.4 (CH2), 28.4 (C(*C*H3)3), 27.7 (CH*C*H2), 26.2 (C*C*H2­CH), 25.8 (C*C*H2CH); *m/z* (ES+) 272 (100), 270 (10), 267 (1) 266 (1); C15H23NO2Na (MNa+) requires 272.1626, found 272.1619. The diene was found to re-aromatise on standing in air to *tert*-butyl {[(*S*)-2,3-dihydro-1*H*-inden-1-yl]methyl}carbamate (**40)**, a white crystalline solid. *R*f (1:1 Et2O : petroleum ether) 0.38; δH (CDCl3, 400 MHz) 7.26–7.16 (4H, m, 4 × aromatic CH), 4.58 (1H, br s, NH), 3.57–3.46 (1H, m, CH), 3.40–3.20 (2H, m, NCH2), 3.02–2.82 (2H, m, CHC*H2*), 2.31–2.18 (1H, m, C*H*H), 1.89–1.78 (1H, m, CH*H*), 1.62 and 1.45 (0.7H and 8.3H, s and s, rotamers, C(CH­3)3); δC (CDCl3, 100 MHz) 156.1 (CO), 144.5 (quat. aromatic); 144.2 (quat. aromatic); 126.9 (aromatic CH), 126.3 (aromatic CH), 124.7 (aromatic CH), 123.8 (aromatic CH), 79.2 (*C*(CH3)3), 45.1 (CH), 44.1 (NCH2), 31.2 (CHCH2), 29.4 (CH2), 28.4 (C(*C*H3)3).

***tert*-Butyl {(*S*)-4-hydroxy-1-[(*S*)-2,3,4,7-tetrahydro-1*H*-inden-1-yl]butyl}carbamate (41)**

To a mixture of freshly-condensed liquid ammonia (20 mL), THF (2 mL) and EtOH (0.5 mL) at −78 °C was added azacycle (+)-**10** (75 mg, 0.40 mmol) in THF (1 mL). Sodium pieces (~10 mg each, *ca*. 120 mg in total) were slowly added until a blue colour persisted. Further additions of sodium were made when the blue colour had dissipated until the blue colour was found to persist again (*ca*. 60 mg). After 4 h TLC analysis indicated complete consumption of starting material. The reaction was quenched by the addition of solid ammonium chloride (1.00 g, 19 mmol). The mixture was allowed to warm to rt over 16 h; the organic residue was extracted with Et2O (3 × 30 mL), dried (MgSO4) and the solvent removed under reduced pressure. Column chromatography (90% Et2O in petroleum ether) gave diene **41** as a yellow oil (37 mg, 56%) that decomposed on standing (over 24 h). *R*f (9:1 Et2O : petroleum ether) 0.20; [α]25D +12.4 (CHCl3, *c* = 1.00); νmax (Nujol) 3482s (OH str), 3311m (NH str), 2924s (CH str), 1685s (C=O str); δH (CHCl3, 400 MHz) 5.77–5.67 (2H, m, 2 × vinylic CH), 4.46–4.21 (1H, m, NH), 3.89–3.77 (1H, m, CH), 3.72–3.62 (2H, m, CH2OH), 2.82–2.49 (5H, m, CH and 2 × C=C-CH2), 2.30–2.12 (3H, m, CH2 and C*H*H), 2.02–1.90 (1H, m, CH*H*), 1.69–1.49 (4H, m, C*H2*C*H2*NCH), 1.39 (9H, s, C(CH3)3); δC (CDCl3, 100 MHz) 156.1 (CO), 134.0 (quat. vinylic), 132.2 (quat. vinylic), 126.7 (vinylic CH), 124.7 (vinylic CH), 79.1 (*C*(CH3)3), 62.6 (CH2OH), 51.6 (NHCH), 50.8 (CH), 34.5 (CH2), 30.5 (CH2), 29.4 (CH2), 28.3 (C(*C*H3)3), 27.7 (CH2).

**References**

1. Pangborn, A. B.; Giardello, M. A.; Grubbs, R. H.; Rosen, R. K.; Timmers, F. J. *Organometallics* **1996,** *15,* 1518–1520. doi:[10.1021/om9503712](http://dx.doi.org/10.1021/om9503712)
2. Gottlieb, H. E.; Kotlyar, V.; Nudelman, A. *J. Org. Chem.* **1997,** *62,* 7512–7515. doi:[10.1021/jo971176v](http://dx.doi.org/10.1021/jo971176v)
3. Hodgson, D. M.; Winning, L. H. *Synlett* **2006,** 2476–2479. doi:[10.1055/s-2006-950418](http://dx.doi.org/10.1055/s-2006-950418)
4. Hodgson, D. M.; Bebbington, M. W. P.; Willis, P. *Org. Biomol. Chem.* **2003,** *1,* 3787–3798. doi:[10.1039/b306717n](http://dx.doi.org/10.1039/b306717n)
5. Kopping, B.; Chatgilialoglu, C.; Zehnder, M.; Giese, B. *J. Org. Chem.* **1992,** *57,* 3994–4000. doi:[10.1021/jo00040a048](http://dx.doi.org/10.1021/jo00040a048)
6. Bebbington, M. W. P. Nitrogen-directed free radical rearrangements. D.Phil. Thesis, University of Oxford, U.K., 2002.
7. Swenton, J. S.; Oberdier, J.; Rosso, P. D. *J. Org. Chem.* **1974,** *39,* 1038–1042. doi:[10.1021/jo00922a005](http://dx.doi.org/10.1021/jo00922a005)
8. Arakawa, Y.; Yasuda, M.; Ohnishi, M.; Yoshifuji, S. *Chem. Pharm. Bull.* **1997,** *45,* 255–259.
9. Hodgson, D. M.; Maxwell, C. R.; Miles, T. J.; Paruch, E.; Matthews, I. R.; Witherington, J. *Tetrahedron* **2004,** *60,* 3611–3624. doi:[10.1016/j.tet.2004.02.055](http://dx.doi.org/10.1016/j.tet.2004.02.055)
10. Hodgson, D. M.; Maxwell, C. R.; Wisedale, R.; Matthews, I. R.; Carpenter, K. J.; Dickenson, A. H.; Wonnacott, S. *J. Chem. Soc., Perkin Trans. 1* **2001,** 3150–3158. doi:[10.1039/b107414h](http://dx.doi.org/10.1039/b107414h)
